# The 8-Hydroxyquinoline Derivatives of 1,4-Naphthoquinone: Synthesis, Computational Analysis, and Anticancer Activity

**DOI:** 10.3390/ijms26115331

**Published:** 2025-06-01

**Authors:** Arkadiusz Sokal, Roman Wrzalik, Małgorzata Latocha, Monika Kadela-Tomanek

**Affiliations:** 1 Department of Organic Chemistry, Faculty of Pharmaceutical Sciences in Sosnowiec, Medical University of Silesia, 4 Jagiellońska Str., 41-200 Sosnowiec, Poland; asokal@outlook.com; 2 Doctoral School, Medical University of Silesia in Katowice, 15 Poniatowskiego Str., 40-055 Katowice, Poland; 3 Silesian Center for Education and Interdisciplinary Research, University of Silesia, 75Pułku Piechoty 1a, 41-500 Chorzów, Poland; roman.wrzalik@us.edu.pl; 4 Department of Cell Biology, Faculty of Pharmaceutical Sciences in Sosnowiec, Medical University of Silesia, 8 Jedności Str., 41-200 Sosnowiec, Poland; mlatocha@sum.edu.pl

**Keywords:** quinoline, 1,4-naphthoquinone, structure analysis, NMR, IR

## Abstract

Anticancer drug design has been reformed by the creation of heterocyclic hybrids. The introduction of a quinoline scaffold affects the activity, toxicity, and bioavailability of new compounds. The aim of this study was to synthesize and evaluate the biological activity of hybrids of 1,4-naphthoquinone with the 8-hydroxyquinoline moiety. The structure of the new compounds was characterized using spectroscopic methods, such as HR-MS, NMR, and IR. The analysis was supplemented by calculated NMR and IR spectra. The physicochemical properties and bioavailability of the compounds were examined using in silico methods. An analysis of reactivity descriptors showed that the compounds are good electron acceptors and exhibit high reactivity. Bioavailability properties confirm that hybrids could be good oral administration drugs. The biological potential of hybrids was examined by designation of the enzymatic conversion rate of the NQO1 protein and in vitro against cancer cell lines with overexpression of the gene encoding the NQO1 protein. The possibility of interaction between the tested ligand and the NQO1 protein was examined by molecular docking methods.

## 1. Introduction

**Figure 1:**
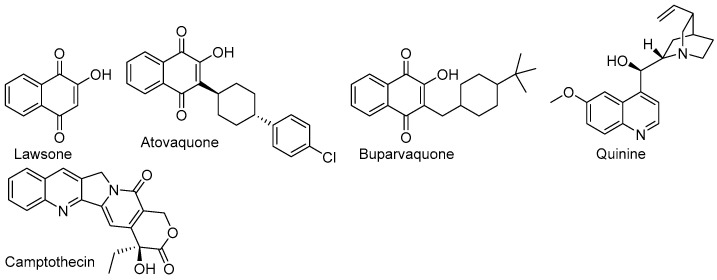
Chemical structure of natural and semi-synthetic compounds.

Compounds with 1,4-naphthoquinone pharmacophore are large groups of natural substances that can be obtained from plants, animals, fungi, and bacteria [1]. One of the best-known 1,4-naphthoquinones is lawsone (Figure 1), which is an extract from *Lawsonia inermis*. Even though the substance was isolated in the 1950s, the properties of the leaves were well-known in ancient times. People in North Africa, the Middle East, and South Asia use the powdered leaves as a brown dye for hair coloring, body paint, and tattoos. Moreover, the brown powder is used to dye silk, wool, and leather [1,2].

Lawsone is characterized by a broad spectrum of pharmacological activities, such as anticancer, antibacterial, antifungal, antiviral, antimalarial, and anti-inflammatory effects [3,4,5,6]. The biological research shows that the compound influences many different enzymes. However, the most important effect seems to be caused by inducing oxidative damage by the production of different types of reactive oxygen species (ROS), which affect DNA mutation [7,8]. This natural compound is also characterized by in vivo genotoxicity, which limits its use in human therapy [2,9].

The most important modification of the lawsone structure is the replacement of the hydroxyl group with an alkoxy group [10]. For example, Durán and co-workers described the series of 2-O-alkyl derivatives, which exhibited high cytotoxicity towards different cancer cell lines, while its activity against non-cancer cell lines was similar to the reference substance, etoposide [11]. The introduction of alkynyloxy or aryloxy substituents at the C2 position of 1,4-naphthoquinone moiety increases the antifungal activity against *S. aureus*, *E. coli*, and *C. albicans* [12,13]. The second possible modification of the lawsone structure is introducing a substituent at the C3 position. This group of compounds includes atovaquone and buparvaquone (Figure 1). Atovaquone is used in the treatment of malaria, toxoplasmosis, and pneumocystis pneumonia, while buparvaquone is used in bovine theileriosis [14]. The analysis of the structure–activity relationship shows that activity depends on the type of substituent at the C2 and C3 positions. The introduction of an ether or thioether group increases antibacterial activity, while substituents with nitrogen atoms, such as amine, alkylamine, arylamine, or N-heterocyclic moieties, increase the anticancer effect [10,11,15,16,17,18].

Alkaloids with a quinoline skeleton are found in many natural products and have been used as a panacea for all ailments. One of the first described compounds with a quinoline moiety was quinine (Figure 1), which was isolated from the bark of a cinchona tree. The antiplasmodial activity of this natural substance led to the development of many analogs exhibiting higher activity and better pharmacokinetic parameters [19,20]. In 1958, camptothecin (Figure 1) was obtained from the break of *Camptotheca acuminate*. The compound exhibits high anticancer potential, but its uses are limited due to high toxicity and low bioavailability. Its semi-synthetic derivatives show better pharmacological properties and are used in the treatment of small-cell and non-small-cell lung cancer, cervical cancer, and ovarian cancer [21,22]. Currently, quinoline drugs are used as broad-spectrum antibiotics, chemotherapeutics, and antimalarial drugs [23,24,25]. Recently, many novel 8-hydroxyquinoline derivatives were tested as potential anticancer drugs because they contain a nitrogen atom and a hydroxyl group, which could create hydrogen bonds with biological targets and chelate metal ions. The introduction of additional substituents at the C5 and C2 positions increases the activity [26,27,28,29,30,31].

The action of a compound in a live organism depends on its pharmacological and pharmacokinetic properties. For this reason, the modification of a well-known pharmacophore by connecting it with another active compound allows the development of hybrid derivatives with improved biological and physicochemical properties [32,33]. The literature described hybrid molecules with 1,4-naphthoquinone moiety. Connecting 1,4-naphthoquinone with betulin, diosgenin, or nucleoside increases the activity of hybrids compared to natural substances. The biological research shows that hybrids activate the mitochondrial apoptosis pathway by interacting with NAD(P)H quinone dehydrogenase 1 (NQO1) enzyme [16,34,35,36].

Continuing our research on hybrid compounds with a 1,4-quinone moiety, we designed and synthesized hybrids of 1,4-naphthoquinone with an 8-hydroxyquinoline moiety. The bioavailability parameters of the obtained derivatives were determined using in silico methods. The anticancer potential of hybrids was tested in vitro against four human cancer cell lines. The obtained hybrids were also tested as a substrate of quinone oxidoreductase 1 (NQO1), and the interaction between this protein and the compounds was examined using the molecular docking method.

## 2. Results and Discussion

### 2.1. Synthesis and Structure Analysis

**Figure 2:**
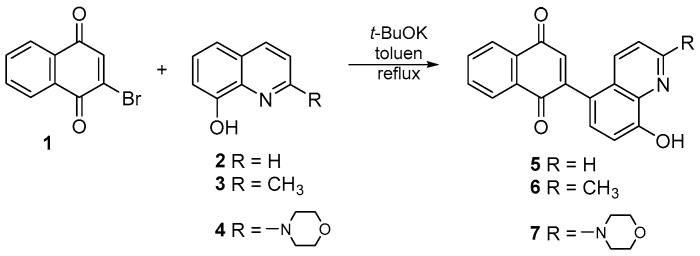
Synthesis route of compounds **5**–**7**.

The literature describes the synthesis of many quinoline compounds that are derivatives of 8-hydroxyquinoline. The reaction occurs in the presence of potassium carbonate and an aprotic solvent such as tetrahydrofuran or dimethylformamide [37,38,39,40]. In this condition, the reaction between 8-hydroxyquinoline derivatives **2**–**4** and 2-bromo-1,4-naphthoquinone **1** does not occur. Treatment of 2-bromo-1,4-naphthoquinone **1** with the corresponding 8-hydroxyquinoline **2**–**4** in the presence of potassium *tert*-butoxide and toluene at reflux allows the obtaining of new derivatives of 1,4-naphthoquinone **5**–**6** (Figure 2).

After purification by column chromatography, pure compounds **5**–**7** were obtained with yields of 62–84%. The derivatives **5**–**7** were the only products of the reaction. The structures of all new compounds were characterized by NMR, FT-IR, Raman, and HR-MS spectroscopy.

#### 2.1.1. HR-MS Spectroscopy

The compounds **5**–**7** were analyzed by high-resolution mass spectroscopy (HR-MS) by direct infusion. The protonated molecule observed at 302.0801 *m/z* (C_19_H_12_NO_3_, calculated as 302.0817 *m/z*) for compound **5**, at 316.0999 *m/z* (C_20_H_14_NO_3_, calculated as 316.0977 *m/z*) for compound **6**, and at 387.1363 *m/z* (C_23_H_19_N_2_O_4_, calculated as 387.1345 *m/z*) for compound **7**. For all tested compounds, peaks were observed with *m/z* values corresponding to the mass of the ion formed by the addition of two protons (Figures S1–S3).

#### 2.1.2. Nuclear Magnetic Resonance Spectroscopy

The chemical structures of derivatives **5**–**7** were analyzed using 1D (^1^H and ^13^C NMR) and 2D (ROESY, HSQC, and HMBC) NMR spectroscopy (Figures S4–S18). Table 1 shows the proton–proton and proton–carbon correlations for compound **5**.

The ROESY spectrum of compound **5** shows that the signals at δ_H_ 8.89 ppm and δ_H_ 8.29 ppm are correlated with the signal at δ_H_ 7.53 ppm (Table 1). The signal at δ_H_ 7.17 ppm is correlated with the signals at δ_H_ 7.53 ppm and δ_H_ 7.50 ppm. The results show that these signals describe protons belonging to the quinoline moiety, while the signals at δ_H_ 8.08 ppm, δ_H_ 7.93 ppm, and δ_H_ 7.12 ppm describe protons belonging to the 1,4-naphthoquinone moiety. The HSQC spectrum allows the assignment of the chemical shifts of carbon signals to proton signals (Table 1). The signal at δ_H_ 10.24 ppm was assigned to the hydrogen atom in the hydroxyl group because no correlation was observed between this peak and the peak of the carbon atom in the HSQC spectrum. The signals of carbon atoms belonging to the quinoline and 1,4-naphthoquinone moieties were assigned based on the correlation observed in the HMBC spectrum. The analysis of this correlation shows that the signal at δ_H_ 8.08 ppm was assigned to protons H5 and H8, while the signal at δ_H_ 7.93 ppm was assigned to H6 and H7 atoms. The signal at δ_H_ 7.12 ppm was assigned to the proton H3 of the 1,4-naphthoquinone moiety. The signal at δ_H_ 8.89 ppm interacts with the signals of carbon atoms at δ_C_ 122.4 ppm and δ_C_ 135.3 ppm, which were assigned to the C3′ and C4′ carbon atoms, respectively. The carbon atom at the C2 position (δ_C_ 148 ppm) was identified based on its correlation with protons at the C6′ and C7′ positions in the quinone moiety. Furthermore, the C5′ carbon (δ_C_ 127.8 ppm) was correlated with the H3′ (δ_H_ 7.53 ppm), H4′ (δ_H_ 8.29 ppm), and H6′ (δ_H_ 7.50 ppm) protons, respectively. The signal at δ_C_ 155.1 ppm was assigned to the carbon atom at the C8′ position based on its correlation with H3′ (δ_H_ 7.53 ppm), H4′ (δ_H_ 8.29 ppm), H6′ (δ_H_ 7.50 ppm), and H7′ (δ_H_ 7.17 ppm).

**Figure 3:**
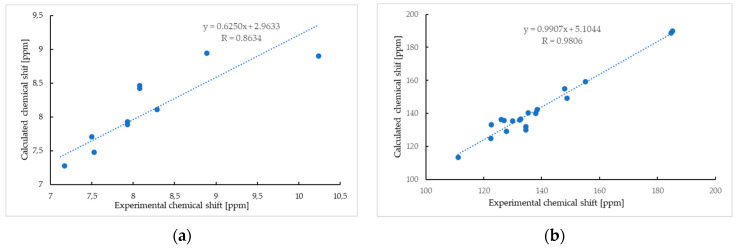
The linear regression between the experimental and calculated ^1^H NMR (**a**) and ^13^C NMR (**b**) chemical shifts of compound **5**.

The analysis was supplemented by computer simulation using the GIAO method [41,42,43]. The ^1^H NMR spectra of compound **5** show good agreement between the experimental and calculated data, with a correlation coefficient of 0.8634 (Figure 3a).

The most important differences between the calculated and experimental ^1^H NMR spectra are observed for the proton of the hydroxyl group (Table 1, Figure 3a). The chemical shifts of the hydroxyl proton in the calculated and experimental spectra are δ_H_ 8.90 ppm and δ_H_ 10.24 ppm, respectively. This effect could be caused by the formation of a hydrogen bond between the hydrogen of the hydroxyl group and other atoms, like oxygen or nitrogen, which influences the chemical shift of the proton [44,45,46]. As seen in Figure 3b, the chemical shift of carbon atoms in the calculated spectrum reproduces the experimental value well, and the correlation coefficient is 0.9806.

The molecular structures of derivatives **6–7** were confirmed by proton–carbon correlation analysis (Tables S1 and S2).

The analysis of the 2D spectra of derivative **6** shows that the proton at δ_H_ 9.82 ppm does not correlate with any carbon or proton, which means that it belongs to the hydroxyl group at the C8′ position of the quinoline moiety (Table S1). The ROESY spectrum shows that the methyl group at the C9′ position of the quinoline ring interacts with the H4′ and H3′ protons. In the HMBC spectrum, the H9′ proton correlates with the carbon atoms at positions C7′ (δ_C_ 110.9 ppm), C3′ (δ_C_ 123.2 ppm), and C2′ (δ_C_ 157.3 ppm) of the quinoline moiety and C3 (δ_C_ 137.8 ppm) of the 1,4-naphthoquinone moiety.

The introduction of a morpholine moiety at the C2′ position of the quinoline moiety significantly influences the chemical shifts of carbon and proton signals (Table S2). The introduction of a morpholine moiety influences the chemical shift of protons in the quinoline moiety. Analysis of proton–proton and proton–carbon correlations shows that the signal at δ_H_ 7.02 ppm was assigned to the proton at the H7′ position of the quinoline moiety, while for derivative **5**, the signal of the proton at H7′ is at δ_H_ 7.17 ppm. Comparing the chemical shift of protons H3′ and H6′ in derivatives **7** and **5** shows that these signals are shifted upfield by Δδ_H_ 0.34 ppm and Δδ_H_ 0.36 ppm, respectively. The group at the C2′ position affects the chemical shift of the signal assigned to the proton at the H3 position of the 1,4-naphthoquinone moiety, which is shifted from δ_H_ 7.09 ppm to δ_H_ 7.02 ppm.

#### 2.1.3. Fourier-Transform Infrared Spectroscopy

**Figure 4:**
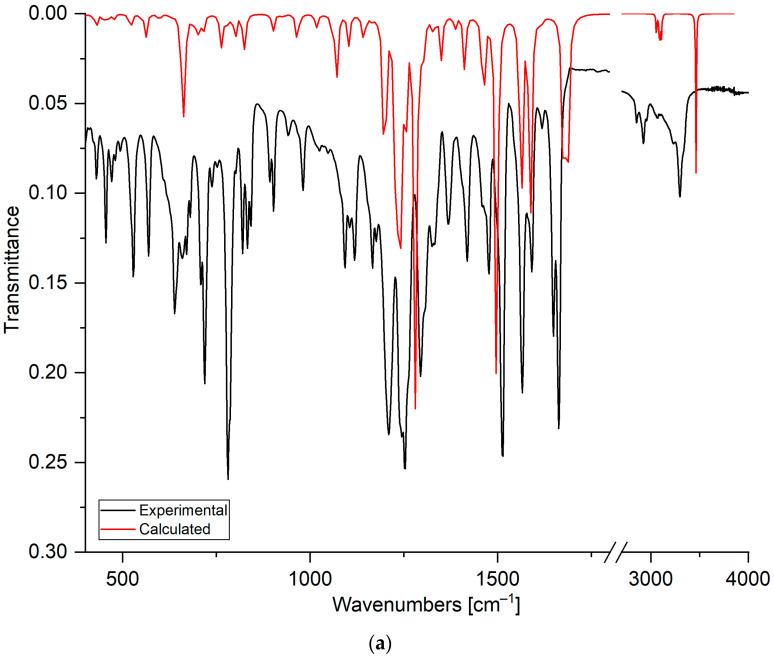

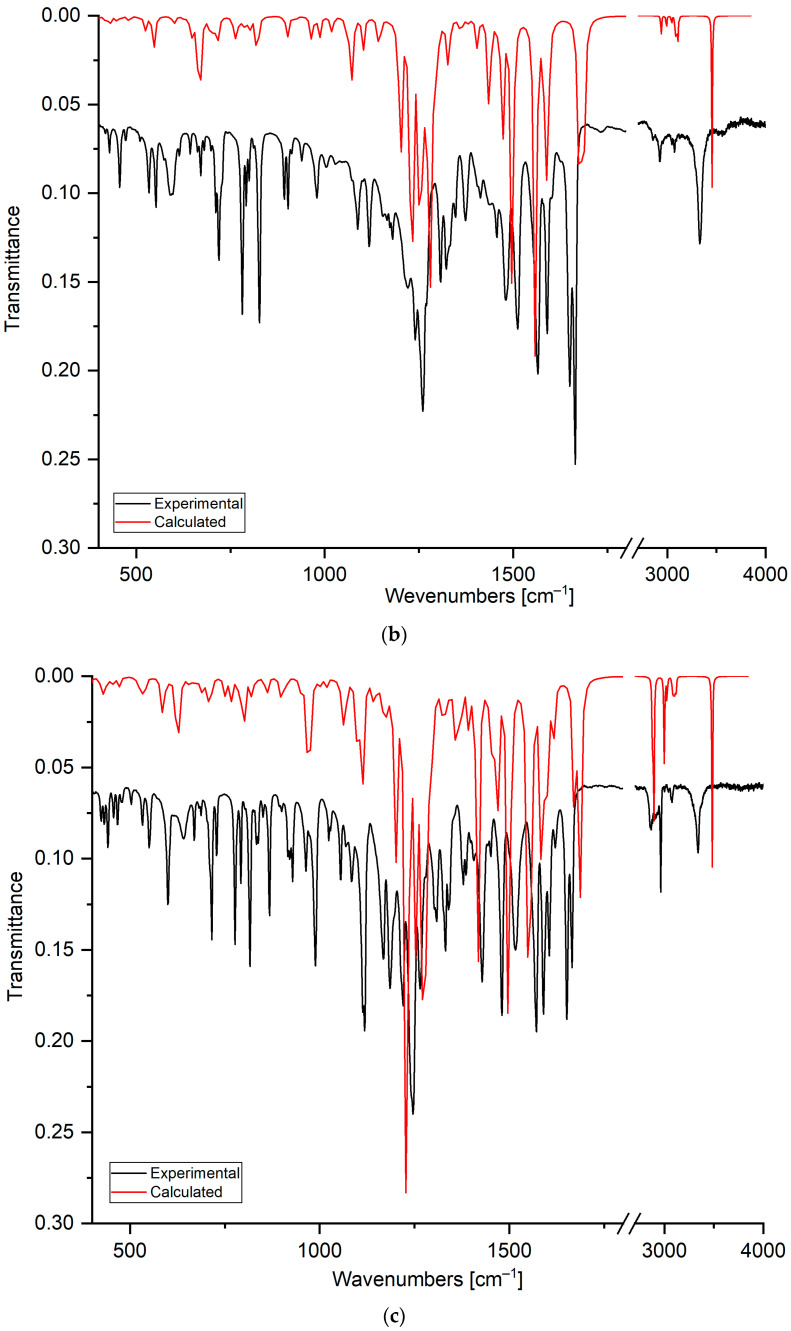
The FT–IR spectra of compounds: (**a**) **5**, (**b**) **6**, and (**c**) **7**. The experimental spectra are shown as black lines, and the calculated spectra as red lines.

The FT-IR spectra were used to confirm the chemical structures of compounds **5**–**7** by analyzing the bands of hydroxyl and carbonyl groups. The bands were assigned by comparing the experimental and calculated FT-IR spectra (Table 2). As seen in Figure 4, the calculated spectrum reproduces the experimental spectrum well.

Comparing the experimental and calculated spectra shows that the stretching vibration in the range from 3340 cm^−1^ to 3298 cm^−1^ is assigned to the hydroxyl group at the C8′ position of the quinoline moiety. The broadening and shifting of the band towards lower wavenumbers compared to the calculated spectrum shows that the hydroxyl group could form a hydrogen bond [44]. The band deformation of the hydroxyl group was observed at 638 cm^−1^, 670 cm^−1^, and 641 cm^−1^ for compounds **5**, **6**, and **7**, respectively. The band in the range from 3077 cm^−1^ to 2853 cm^−1^ is assigned to the stretching vibration of the C-H bond in the quinoline and 1,4-naphthoquinone moieties.

The stretching vibrations of the carbonyl groups are observed in the ranges from 1665 cm^−1^ to 1649 cm^−1^ and from 1678 cm^−1^ to 1673 cm^−1^ in the experimental and calculated spectra, respectively. For compounds **5**–**7**, in the experimental spectra, the carbonyl bands are split into two separate bands. However, in the calculated spectra, this splitting is observed only for derivative **7**. Analysis of the calculated spectra allows the assignment of asymmetric vibrations at higher wavenumbers, mainly to the carbonyl group at the C1 position. Symmetric stretching vibrations are assigned mainly to the carbonyl group at the C4 position. This result suggests that the carbonyl group could create the hydrogen bond [47,48].

The stretching vibrations of carbon–carbon bonds are observed as the wide band in the ranges from 1566 cm^−1^ to 1565 cm^−1^ and from 1583 cm^−1^ to 1588 cm^−1^ in the experimental and calculated spectra, respectively. In the experimental spectra, in the range from 1515 cm^−1^ to 1477 cm^−1^, the bands belong mainly to the deformation vibrations of the C-H group in the naphthoquinone moiety, while the band at 1412–1428 cm^−1^ is usually assigned to the deformation vibration of the C-H group in the quinoline moiety. As seen in Table 2, the bands below 1400 cm^−1^ are assigned to the deformation vibrations of C-C and C-H bonds in both moieties of the compound.

### 2.2. Analysis of Physicochemical Properties

The physicochemical properties influence the fate of a drug in a living organism. The relationship between physicochemical properties and drug absorption after oral administration was first described by Lipinski in 1997. Later studies showed that the bioavailability of a potential drug depends on many properties, such as molecular weight, lipophilicity, topological surface area, and the number of donors and acceptors of hydrogen bonds [49,50]. Table 3 shows the biodistribution parameters of tested compounds **5**–**7**, which were determined using the software available online [51,52].

**Figure 5:**
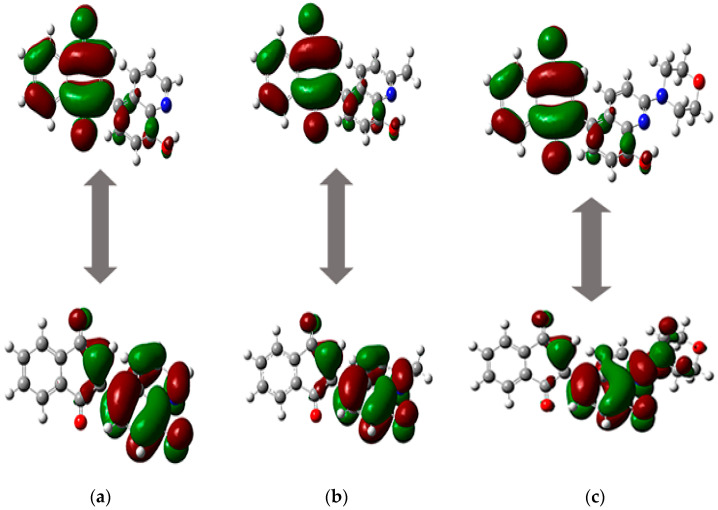
The HOMO and LUMO orbitals of compounds (**a**) **5**, (**b**) **6**, and (**c**) **7**.

Compounds **5**–**7** are characterized by moderate water solubility, which depends on the substituent at the C2′ position of the quinoline moiety, and the order is as follows: morpholine (**7**) < methyl (**6**) < hydrogen (**5**). The same correlation is observed for lipophilicity (logP). Comparing lipophilicity and solubility shows that compounds **5**–**7** can well penetrate biological barriers through passive transport [50,53]. The tested derivatives **5**–**7** also meet all Lipinski’s and Veber’s rules, which means that they should be characterized by good oral availability. The in silico oral administration is described by the Caco-2 permeability (logPapp) and human intestinal absorption (HIA). Compounds **5**–**7** should have good absorption through the intestine because the values of HIA and logPapp are in the ranges from 99% to 97% and from 1.214 to 1.342, respectively. The tested derivatives **5**–**7** may be transported by the skin because the value of logKp is lower than −2.5. After absorption of the drug, the most important parameter is the volume of distribution (VDss), which describes the distribution of the drug from blood to tissue. The determined in silico values show that the concentration of compounds in tissue should be higher than in plasma. The group at the C2′ position of the quinoline moiety influences the penetration of the compound into tissue. The introduction of a morpholine group (**7**) increases the concentration of the compound in tissue by almost twofold compared to the unsubstituted compound (**5**). Compounds are transported to the brain via the bloodstream. The important parameters determining the distribution of a drug in the brain and the central nervous system (CNS) are logBB and logPS. Analysis of these parameters shows that derivatives **5**–**7** will penetrate weakly across the blood–brain barrier and to the CNS. Therefore, 1,4-quinone derivatives **5**–**7** exhibit low neurotoxicity. The metabolism of a drug in silico is described through its interaction with cytochrome P450. As seen in Table 4, the 1,4-quinone derivatives **5**–**7** can interact with cytochrome P450, and they can be transformed into other compounds. The metabolism of the drug depends on its reactivity, which is described by the reactivity descriptors [49,54]. The reactivity properties of the drug are usually designated by the energy of HOMO and LUMO orbitals [55,56]. The molecular orbitals of compounds **5**–**7** were designated using the calculated structures. As seen in Figure 5, for all ligands, the HOMO orbital is localized near the quinoline moiety and the 1,4-quinone unit, while the LUMO orbital is mainly near the 1,4-naphthoquinone moiety and the benzene ring of the quinoline moiety. The overlap of HOMO and LUMO orbitals influences the mobility of electrons.

The energy of frontier orbitals depends on the type of substituent at the C2 position of quinoline moiety, and the order is as follows: hydrogen (**5**) < methyl (**6**) < morpholine (**7**) (Table 3). The energy gap (ΔE) between the HOMO and LUMO orbitals is in the range from 2.491 eV to 2.743 eV. A low ΔE value means that compounds **5**–**7** are highly reactive and can be electron donors or acceptors [57,58]. The energy gap was used to determine the reactivity descriptors of molecules, such as the ionization potential (I), electron affinity (A), hardness (η), chemical potential (µ), electronegativity (χ), and electrophilicity index (ω) (Table 4). Analysis of the ionization potential (I) and electron affinity (A) shows that compounds **5**–**7** have a stronger tendency to gain electrons than remove them. Molecules **5**–**7** are good electron acceptors and are characterized by high reactivity because the η and ω values range from 1.246 eV to 1.371 eV and from 7.227 eV to 7.331 eV, respectively (Table 4) [49].

**Figure 6:**
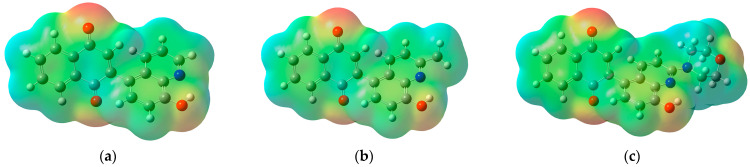
The molecular electrostatic potential maps of derivatives (**a**) **5**, (**b**) **6**, and (**c**) **7**.

Analysis of molecular electrostatic potential (MEP) maps allows the designation of the fragments of molecules that could interact with nucleophilic and electrophilic compounds (Figure 6).

As seen in Figure 6, the most electrophilic areas (blue) of compounds **5**–**7** are localized near the hydrogen atoms of the 1,4-naphthoquinone and quinoline moieties. Additionally, in the MEP map of compound **7**, an electrophilic area near the hydrogen atom of the morpholine moiety can be observed (Figure 6c). The nucleophilic areas (red) are localized near the oxygen atoms of the carbonyl and hydroxyl groups. The high reactivity of derivatives **5**–**7** is due to the even arrangement of nucleophilic and electrophilic regions throughout the whole molecule.

Analysis of reactivity descriptors shows that the probability of transformation of the compound into other molecules is high. For this reason, the in silico extraction and toxicity assay parameters do not represent the fate of a drug in a living organism.

### 2.3. Biological Activity

#### 2.3.1. NQO-1 Activity

The NAD(P)H-quinone oxidoreductase 1 (NQO1) enzyme is a flavoenzyme that catalyzes the two-electron reduction of quinone using NADPH. The products of the redox reaction are hydroquinone and reactive oxygen species (ROS), which damage the DNA, leading to cell death. In normal cells, the enzyme level is hardly detectable, while the overexpression of the gene encoding the NQO1 protein is detected in many types of cancer cells, such as breast, colon, cervix, lung, and pancreas. The interesting targets of cancer therapy are quinone compounds that exhibit high activity against the NQO1 enzyme [59,60].

The 1,4-quinone derivatives **5**–**7** and 2-bromo-1,4-naphthoquinone **1** were tested as substrates of the NQO1 enzyme using a method described in the literature [61,62]. As a reference substance, β-lapachone (β-Lap) was used, which is an NQO1-directed antitumor agent. The ability of the compounds to be a substrate of the NQO1 protein was examined using the oxidation of NADPH to NADP+. The enzymatic conversion rates of NQO1 for the tested compounds are presented in Table 5.

In the series of tested compounds, the lowest value of the enzymatic conversion rate of the NQO1 enzyme was obtained for 2-bromo-1,4-naphthoquinone **1**. The introduction of the quinone moiety increases the enzymatic conversion rate. However, the activity of compounds **5**–**7** depends on the substituent at the C2′ position of quinoline, with the order as follows: methyl group (**6**) > morpholine group (**7**) > hydrogen atom (**5**). Compound **6** with a methyl group at the C2′ position was identified as the NQO1 substrate with the highest efficiency, which is better than β-lapachone.

#### 2.3.2. Anticancer Activity In Vitro

The anticancer potential of compounds **1** and **5**–**7** was tested against four human cancer cell lines, which show overexpression of the gene encoding the NQO1 protein. As reference substances, β-lapachone and cisplatin were used. The results are presented in Table 6.

The analysis of the structure–activity relationship shows that the replacement of the bromine atom by the 8-hydroxyquinoline moiety increases the anticancer activity against all tested cell lines (Table 6). In the series of tested hybrids, the observed trend of activity against all tested cell lines is that compound **6**, with a methyl group at the C2′ position of the quinoline moiety, shows the highest cytotoxicity, while the introduction of the morpholine moiety reduces the activity compared to derivative **5** with a hydrogen atom at this position.

Comparing the activity of 1,4-naphthoquinone compounds **5**–**7** and cisplatin shows that hybrids **5**–**7** exhibit higher cytotoxicity than the reference substance against lung (A549) and breast (MCF-7) cell lines. However, only against A549 cells do they show higher activity than β-lapachone.

As seen in Table 6, the activity of hybrids **5**–**7** depends on the type of cancer cell line. For this reason, the cytotoxicity of the derivatives was compared with the level of the gene encoding the NQO1 protein and the enzymatic conversion rate of NQO1. According to the Human Protein Atlas, the highest value of the nTPM (transcripts per million) index is determined for lung (A549) and amelanotic melanoma (C-32) cell lines [63,64]. Comparing the IC_50_ and nTPM values, it can be observed that the activity correlates well with the level of expression of the gene encoding the NQO1 protein, and the order is as follows: A549 > C-32 > MCF-7 > Colo-829. Comparing the activity and enzymatic conversion rate of NQO1 shows that derivative **6** exhibits the highest activity and enzymatic conversion rate.

In our previous study, we obtained hybrids of 1,4-naphthoquinone with quinoline, which are connected by an oxygen linker [18]. Both groups of derivatives exhibit the highest activity against the lung cancer cell line (A549). Compound **5** exhibits slightly lower activity than 2-chloro-3[(quinolin-8-yl)oxy]naphthalene-1,4-dione against A549 cells [18]. Comparing the IC_50_ value against these cancer cells shows that derivatives **6**–**7** are characterized by higher activity than the hybrids with an oxygen linker. The obtained results suggest that the hydroxyl group at C8′ and the substituent at the C2′ position of the quinoline moiety influence the anticancer activity.

### 2.4. Molecular Docking Analysis

The interaction between the NQO1 protein and the tested compounds was analyzed using the molecular docking method. As seen in Table 7, compounds with a quinoline moiety (**5**–**7**) show lower binding energy values than 2-bromo-1,4-naphthoquinone **1,** which means that they interact more strongly with the active center of the protein. Comparing the values of ΔG and the enzymatic conversion rate of NQO1 shows that molecular docking reproduces the experimental results well.

**Figure 7:**
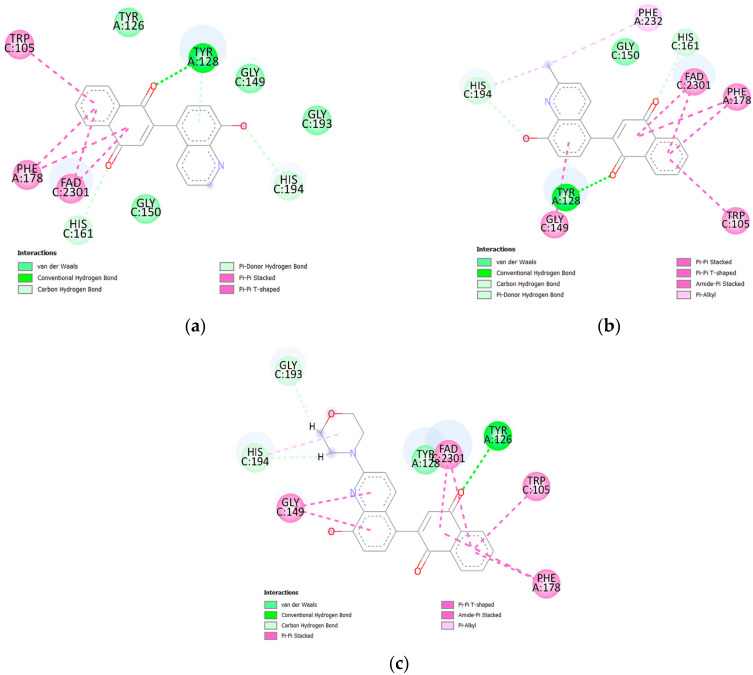
Docking poses of NQO1 protein complex with compounds (**a**) **5**, (**b**) **6**, and (**c**) **7**.

The molecular arrangement of the active center and data about the type and length of bindings between the compounds and protein are presented in Figure 7 and Table S3, respectively.

Ligands **5** and **6** create hydrogen bonds with tyrosine (TYR128) and histidine (HIS161 and HIS194). However, the carbonyl groups at the C1 and C4 positions of the 1,4-naphthoquinone moiety interact with TYR 128 and HIS161, respectively (Figure 7). The hydroxyl group of the quinoline moiety creates a weak H-bond with HIS164. In both ligand–protein complexes, the 1,4-naphthoquinone moiety creates hydrophobic interactions with tryptophan (TRP105), phenylalanine (PHE178), and the FAD cofactor. In the ligand **6**–protein complex, hydrophobic interactions are also observed between the quinoline moiety and two amino acids, glycine (GLY149) and phenylalanine (PHE232). The compound with the morpholine group, **7,** shows a different arrangement in the active site of the protein. For this ligand, only the carbonyl group at the C4 position of the 1,4-naphthoquinone moiety forms a hydrogen bond with tyrosine (TYR126). Glycine (GLY193) and histidine (HIS194) form H-bonds with the morpholine moiety. In this case, the hydroxyl group does not form a hydrogen bond with any amino acid. Analysis of molecular docking shows that the enzymatic conversion rate depends on the arrangement of the 1,4-quinone moiety in the active site of the NQO1 protein.

The arrangement of ligand **6** in the active site of the NQO1 protein was compared with the arrangement of 2-chloro-3-[(2-methylquinolin-8-yl)oxy]naphthalene-1,4-dione [18]. In both cases, the ligands form hydrogen bonds with tyrosine (TYR128) and hydrophobic interactions with tryptophan (TRP105), phenylalanine (PHE178), and the cofactor FAD. The analysis shows that the arrangement of these two ligands in the active site of the protein is similar and does not depend on how the two fragments are connected.

## 3. Materials and Methods

### 3.1. Physical Characterization

Melting points were measured by the Electrothermal IA 9300 melting point apparatus. The high-resolution mass spectra (HR-MS) were determined using the Bruker Impact II instrument (Brucker Analytische Messtechnik GmbH, Rheinstetten, Germany). The spectra were visualized using the Bruker Compass DataAnalysis 4.3 software. The experimentally determined molecular weight was compared with the theoretical value obtained by the Exact Mass Calculator available online [65].

Nuclear magnetic resonance spectra (NMR) were recorded using a Brucker Avance 600 spectrometer (Bruker, Billerica, MA, USA). The sample was prepared by dissolving 10 mg of the compound in 0.6 mL of hexadeuterodimethyl sulfoxide (DMSO-d6). As a reference substance, we used dimethyl sulfoxide (DMSO). The ^1^H and ^13^C NMR spectra were recorded at 600 MHz and 150 MHz frequencies, respectively. Chemical shifts (δ) are reported in ppm and coupling constants (*J*) in Hz. The multiplicity is marked as singlet (s), broad singlet (bs), doublet (d), or multiplet (m). The space correlations were examined by the ROESY (rotating-frame nuclear Overhauser effect spectroscopy) spectrum. The correlation between proton and carbon was analyzed using HSQC (heteronuclear single quantum coherence) and HMBC (heteronuclear multiple-bond correlation) spectra. The obtained spectra were analyzed using the MestReNova 15.0 software.

Fourier-transform infrared spectra (FT-IR) were determined using the FT-IR spectrometer Nicolet iS50 (Thermo Fisher Scientific, Waltham, USA, USA) equipped with the attenuated total reflection (ATR) diamond accessory MIRacle (PIKE Technology, Fitchburg, WI, USA). The spectra were accumulated with a resolution of 2 cm^−1^ (digital resolution 0.482 cm^−1^) in the spectral range of 400–4000 cm^−1^. The OriginPro 9.1 software was used to analyze the spectra.

### 3.2. Synthesis of Compounds ***5***–***7***

The 2-bromo-1,4-naphthoquinone **1** (0.1 g; 0.422 mmol) was dissolved in 2 mL of toluene and potassium *tert*-butoxide (1.5 eqv; 0.633 mmol; 0.071 g) was added. After 15 min at room temperature, the corresponding 8-quinolinol **2**–**4** (1.5 eqv; 0.633 mmol) was added, and the reaction mixture was heated to boiling temperature. The progress of the reaction was controlled using thin-layer chromatography (TLC). After 24 h, the reaction mixture was concentrated under reduced pressure. The crude products **5**–**7** were purified by column chromatography using chloroform/ethanol (40:1, *v*/*v*) as the eluent to give the following:

*2-(8-hydroxyquinolin-5-yl)-1,4-naphthalenedione* **5** yield 71%; mp. 239–240 °C; ^1^H NMR (600 MHz, DMSO-d6) δ, ppm: 7.12 (s, 1H, H3), 7.17 (d, J = 7.2 Hz, 1H, H7′), 7.50 (d, J = 7.2 Hz, 1H, H6′), 7.53 (d, J = 7.8 Hz, 1H, H3′), 7.93 (bs, 2H, H6, H7), 8.08 (bs, 2H, H8, H5), 8.29 (d, J = 7.8 Hz, 1H, H4′), 8.89 (s, 1H, H2′), 10.24 (bs, 1H, OH); ^13^C NMR (150 MHz, DMSO-d6) δ, ppm: 111.1 (C7′), 122.4 (C3′), 122.6 (C4A′), 126.0 (C5), 127.0 (C8), 127.8 (C5′), 130.0 (C6′), 132.4 (C10), 132.7 (C9), 134.6 (C6, C7), 135.3 (C4′), 138.0 (C3), 138.5 (C8A′), 148.0 (C2), 148.7 (C2′), 155.1 (C8′), 184.7 (C5), 185.1 (C8); IR (ν_max_ cm^−1^, ATR): 3298, 1662, 1649, 1419, 1118, 712; ESI-HRMS *m/z* [M+1]^+^ calcd for C_19_H_12_NO_3_^+^ 302.0817, found 302.0801.

*2-(8-hydroxy-2-methylquinolin-5-yl)-1,4-naphthalenedione* **6** yield 84%; mp. 221–222 °C; ^1^H NMR (600 MHz, DMSO-d6) δ, ppm: 2.71 (s, 3H, CH_3_), 7.09 (s, 1H, H3), 7.17 (d, J = 7.8 Hz, 1H, H7′), 7.40 (s, 1H, H6′), 7.41 (s, 1H, H3′), 7.93 (m, 2H, H6, H7), 8.07 (m, 2H, H8, H5), 8.15 (d, J = 8.4 Hz, 1H, H4′), 9.82 (bs, 1H, OH); ^13^C NMR (150 MHz, DMSO-d6) δ, ppm: 25.1 (CH_3_), 110.9 (C7′), 122.6 (C4A′), 123.2 (C3′), 126.0 (C5), 126.1 (C5′), 127.0 (C8), 128.9 (C6′), 132.4 (C10), 132.7 (C9), 134.6 (C6, C7), 135.3 (C4′), 137.8 (C3), 137.8 (C8A′), 148.1 (C2), 154.3 (C8′), 157.3 (C2′), 184.7 (C5), 185.2 (C8); IR (ν_max_ cm^−1^, ATR): 3330, 1664, 1650, 1412, 1118, 712; ESI-HRMS *m/z* [M+1]^+^ calcd for C_20_H_14_NO_3_^+^ 316.0977, found 316.0999.

*2-(8-hydroxy-2-morpholinoquinolin-5-yl)-1,4-naphthalenedione* **7** yield 62%; mp. 225–226 °C; ^1^H NMR (600 MHz, DMSO-d6) δ, ppm: 3.75 (m, 8H, CH_2_), 7.02 (d, J = 7.8 Hz, 1H, H7′), 7.04 (s, 1H, H3), 7.14 (d, J = 7.8 Hz, 1H, H6′), 7.19 (d, J = 9.0 Hz, 1H, H3′), 7.92 (m, 2H, H6, H7), 7.96 (d, J = 9.0 Hz, 1H, H4′), 8.07 (m, 2H, H8, H5), 9.08 (bs, 1H, OH); ^13^C NMR (150 MHz, DMSO-d6) δ, ppm: 45.5 (CH_2_), 66.6 (CH_2_), 110.7 (C3′), 110.8 (C3), 121.9 (C5′), 122.6 (C4A′), 125.3 (C6′), 126.0 (C5), 127.0 (C8), 132.4 (C10), 132.7 (C9), 134.5 (C6, C7), 136.8 (C4′), 137.2 (C8A′), 137.4 (C7′), 148.3 (C2), 152.7 (C8′), 156.3 (C2′), 184.8 (C5), 185.2 (C8); IR (ν_max_ cm^−1^, ATR): 3340, 1665, 1652, 1428, 1118, 729; ESI-HRMS *m/z* [M+1]^+^ calcd for C_23_H_19_N_2_O_4_^+^ 387.1345, found 387.1363.

### 3.3. Computational Details

The molecular structures of compounds **5**–**7** were determined using methods described in the literature [16,41,49,62]. The calculated structure of molecules was used to determine the theoretical nuclear magnetic resonance (NMR) and vibrational (IR) spectra [43,66]. The theoretical wavenumber in IR spectra was scaled by a factor of 0.964 [67,68]. The NMR spectra were calculated by the gauge-independent atomic orbital (GIAO) method [41,42]. The optimized ligand structure was used to designate the molecular orbitals and molecular electrostatic potential maps. The visualization of calculated results was performed in the GaussView, Version 5 software [69].

### 3.4. The ADMET Study

The ADMET parameters were determined using software available online, such as pkCMS and SwissADME [50,51,70,71].

### 3.5. Enzymatic Study

The enzymatic assay was determined using the previously described methods [62,72,73]. As a reference substance, β-lapachone was used. Briefly, a solution of DT-diaphorase (NQO1, EC 1.6.5.5, human recombinant, Sigma-Aldrich, St. Louis, MO, USA) in potassium phosphate buffer (50 mmol/L) was added to the 96-well plate (Nunc Thermo Fisher Scientific, Waltham, MA, USA). Compounds **5**–**7** (0.01 µmol) were dissolved in 1 mL of dimethyl sulfoxide (DMSO), and the obtained solution (2 µL) was added to each well. After 2 min of shaking, the 40 µL of water solution of nicotinamide adenine dinucleotide phosphate (NADPH) (400 µmol/L) was added to each well. A BioTek 800TS microplate reader (BioKom, Poland) was used to measure the absorption of NADPH at a wavelength of 340 nm. The data were recorded at 10 s intervals for 10 min at 25 °C. NADPH oxidation rates were compared with reactions lacking compound. Initial velocities were calculated, and data were expressed as µmol NADPH/µmol NQO1/min. For each of compounds **5**–**7** and the reference substance, the measurements were performed three times.

### 3.6. Anticancer Study

Compounds **1** and **5**–**7**, β-lapachone, and cisplatin were tested for in vitro anticancer activity against four human cancer cell lines, such as lung (A549, ATCC, Rockville, MD, USA), breast (MCF-7, ATCC, Rockville, MD, USA), and melanoma (C32 and Colo-829, ATCC, Rockville, MD, USA). The cultured cells were kept at 37 °C and 5% CO_2_. The cells were seeded (5 × 10^4^ cells/well/100 mL DMEM supplemented with 10% FCS and streptomycin/penicillin) using 96-well plates (Nunc Thermo Fisher Scientific, Waltham, MA, USA). The tested compounds with a concentration of 1–100 µg/mL DMSO were incubated with the cancer cells for 72 hrs. The WST-1-formazan (proliferation reagent WST-1 assay, Roche Diagnostics, Mannheim, Germany) was detected using a microplate reader at 450 nm. Results were expressed as a mean value of at least three independent experiments performed in triplicate.

### 3.7. Molecular Docking Study

The optimized structures of derivatives **5**–**7** were used as ligands in the molecular docking study. The crystal structure of DT-diaphorase (NQO1), which was identified as 2F1O, was downloaded from the Protein Data Bank (PDB) [74]. The protein was crystallized as a cocrystal structure with the flavin adenine dinucleotide (FAD) molecule, which is a cofactor. The molecular docking analysis was performed using the AutoDock Vina software [75]. The grid center of Vina docking was selected as the center of the reference ligands that accompanied the downloaded protein complexes (x = 11.456160; y = 12.047880; z = −5.676920). The grid size was set to 15 Å × 15 Å × 15 Å, which is large enough to cover the entire target active site. Default values of all other parameters were used, and the complexes were submitted to 8 genetic algorithm runs. All obtained results were visualized using the BIOVIA Discovery Studio software package [76].

## 4. Conclusions

The new type of quinoline-1,4-naphthoquinone hybrids **5**–**7** was obtained in the presence of potassium tert-butoxide and toluene. The structures of compounds **5**–**7** were characterized using different spectroscopic methods, including HSQC and HMBC, which allowed the analysis of the hydroxyl group at the C8′ position of the quinoline moiety. The bioavailability of the compounds **5**–**7** was determined by in silico methods. Analysis of Lipinski and Veber’s rules showed that these derivatives could be orally administered drugs. Moreover, they could be well absorbed from the gastrointestinal system, and their concentration in tissue should be higher than in blood. Reactivity descriptors were determined based on the energy of HOMO and LUMO orbitals, which showed that the tested hybrids **5**–**7** are highly reactive and have a stronger tendency to gain electrons than to remove them. The compounds **5**–**7** are good substrates for the NQO1 protein. The in vitro anticancer activity showed that derivatives **5**–**7** possessed the highest activity against the lung cancer cell line, which was characterized by the highest nTPM value in the series of tested cell lines. The activity depended on the type of substituent at the C2′ position of the quinoline ring. The same correlation was observed for the scoring value obtained for the ligand–protein complex.

## Figures and Tables

**Table 1:** The chemical shift of compound **5**.

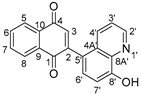								
Proton	^1^H NMR [ppm]	^1^H NMR_calc_ [ppm]	ROESY	HSQC	Carbon	^13^C NMR [ppm]	^13^C NMR_calc_ [ppm]	HMBC
OH	10.24	8.90						
H2′	8.89	8.94	H2′(8.89)-H3′(7.53)	H2′(8.89)-C2′(148.7)	C2′	148.7	149.2	H2′(8.89)-C3′(122.4) H2′(8.89)-C4′(135.3) H2′(8.89)-C8A′(138.5)
H4′	8.29	8.11	H4′(8.29)-H3′(7.53)	H4′(8.28)-C4′(135.3)	C4′	135.3	140.2	H4′(8.89)-C3′(122.4) H4′(8.89)-C8A′(138.5) H4′(8.89)-C2′(148.7) H4′(8.89)-C5′(127.8) H4′(8.89)-C8′(155.1)
H5 H8	8.08	8.46 8.42	H8(8.08)-H6(7.93)	H5/H8(8.08)-C5(126.0) H5/H8(8.08)-C5(127.0)	C5 C8	126.0 127.0	135.7 136.3	H5/H8(8.08)-C6(134.6) H5/H8(8.08)-C10(132.4)
H6 H7	7.93	7.93 7.89	H7(7.93)-H8(8.08)	H6/H7(7.93)-C6/C7(134.6)	C6 C7	134.6	132.0 130.1	H6/H7(7.93)-C8(127.0) H6/H7(7.93)-C5(126.0) H6/H7(7.93)-C10(132.4) H6/H7(7.93)-C9(132.7)
H3′	7.53	7.48	H3′(7.53)-H4′(8.28) H3′(7.53)-H2′(8.89)	H3′(7.53)-C3′(122.4)	C3′	122.4	124.8	H3′(7.53)-C2′(148.7) H3′(7.53)-C5′(127.8) H3′(7.53)-C8′(155.1)
H6′	7.50	7.71	H6′(7.50)-H7′(7.17)	H6′(7.50)-C6′(130.0)	C6′	130.0	135.5	H6′(7.50)-C5′(127.8) H6′(7.50)-C2(148.0) H6′(7.50)-C8′(155.1) H6′(7.50)-C8A′(138.5)
H7′	7.17	7.28	H7′(7.17)-H6′(7.50)	H7′(7.17)-C7′(111.1)	C7′	111.1	113.5	H7′(7.17)-122.6 H7′(7.17)-155.1 H7′(7.17)-138.5
H3	7.12	6.95	-	H3(7.12)-C3(138.0)	C3	138.0	140.1	H3(7.12)-C4A′(122.6) H3(7.12)-C10(132.4) H3(7.12)-C2(148.0) H3(7.12)-C4(184.7)
					C4A′	122.6	133.1	C4A′(122.6)-H3(7.12) C4A′(122.6)-H7′(7.17)
					C5′	127.8	129.0	C5′(127.8)-H4′(8.29) C5′(127.8)-H3′(7.53) C5′(127.8)-H6′(7.50)
					C10	132.4	136.1	C10(132.4)-H7′(7.12) C10(132.4)-H6/H7(7.93) C10(132.4)-H5/H8(8.08)
					C9	132.7	136.6	C9(132.7)-H6/H7(7.93)
					C8A′	138.5	142.2	C8A′(138.5)-H2′(8.89) C8A′(138.5)-H4′(8.29) C8A′(138.5)-H6′(7.50) C8A′(138.5)-H7′(7.17)
					C2	148.0	154.9	C2(148.0)-H6′(7.50) C2(148.0)-H3(7.12)
					C8′	155.1	159.3	C8′(155.1)-H4′(8.29) C8′(155.1)-H3′(7.53) C8′(155.1)-H6′(7.50) C8′(155.1)-H7′(7.17)
					C4	184.7	188.6	C4(184.7)-H3(7.12)
					C1	185.1	189.9	-

**Table 2:** Experimental and calculated wavenumber [cm^−1^] and band assignments for compounds **5**–**7**.

Band	Wavenumber [cm^−1^]					
	5		6		7	
	Exp.	Calc.	Exp.	Calc.	Exp.	Calc.
v O-H	3298	3463	3330	3455	3340	3483
v C-H	2953–2853	3108–3054	3072–2853	3108–2940	3077–2855	3118–2893
v_as_ C=O	1662	1689	1664	1689	1665	1687
V_syn_ C=O	1649	1673	1650	1673	1652	1669
v C-C naph	1591–1566	1588	1590–1565	1589	1590	1583
δ C-H quinone	1514–1477	1496–1465	1510–1479	1496–1472	1515–1480	1496–1470
δ C-H quinone	1419	1411	1412	1403	1428	1418
δ C-C quinone	1369	1350	1374	1357	1385–1379	1357
δ C-C naphtho	1294	1280	1307	1280	1265	1270
δ C-C quinone	1254–1240	1242	1260–1220	1248–1233	1246–1220	1253–1227
δ C-H naphto	1211–1167	1195–1141	1180–1154	1206	1186–1169	1201
δ C-C nap	1118	1103	1118	1102	1118–1114	1114
δ C-C quinone	1092	1072	1088	1072	1084	1066
δ C-H quinone	843–781	825–802	902–893	902	867–816	865–821
δ C-C	719–708	717–702	719–698	717–697	729–715	715
δ O-H	680–638	663	680–662	670	639	629
δ C-C	569–529	563–524	552–534	544–524	550–530	530

**Table 3:** The ADMET parameters of compounds **5–7**.

Parameter	5	6	7
logS	−4.05	−4.36	−4.45
logP	2.21	2.49	2.71
M	301.3	315.32	386.4
TPSA	64.26	67.26	79.73
nHA	4	4	5
nHD	1	1	1
nRT	1	1	2
logPapp	1.250	1.214	1.342
HIA [%]	98	97	99
logKp	−2.738	−2.747	−2.766
logBB	−0.126	−0.143	−0.499
logPS	−2.000	−1.926	−2.231
logVDss	0.086	0.076	0.166
CYP3A4 substrate	Yes	Yes	Yes
CYP3A4 inhibitor	No	No	No

**Table 4:** The reactivity descriptors of compounds **5**–**7**.

Descriptor	5	6	7
E_HOMO_ [eV]	−5.855	−5.772	−5.496
E_LUMO_ [eV]	−3.113	−3.069	−3.004
ΔE [eV]	2.743	2.704	2.491
I [eV]	5.855	5.772	5.496
A [eV]	3.113	3.069	3.004
η [eV]	1.371	1.352	1.246
µ [eV]	−4.484	−4.421	−4.250
χ [eV]	4.484	4.421	4.250
ω [eV]	7.331	7.227	7.249

**Table 5:** The enzymatic conversion rate of NQO1 for compounds **1, 5**–**7,** and β-lapachone (β-Lap).

Ligand	Enzymatic Conversion Rate of NQO1 [µmol NADPH/µmol NQO1/min]
**1**	595 ± 50
**5**	782 ± 42
**6**	1026 ± 69
**7**	851 ± 54
β-Lap	985 ± 45

**Table 6:** The anticancer activity of compounds **1**, **5**–**7**, β-lapachone**,** and cisplatin.

Compounds	Cell Lines/IC_50_ ± SD [µM]			
	A549	MCF-7	C-32	Colo-829
**1**	2.33 ± 0.28	26.34 ± 1.51	7.45 ± 0.08	86.93 ± 2.07
**5**	1.08 ± 0.24	14.08 ± 0.88	3.57 ± 0.02	23.69 ± 2.90
**6**	0.89 ± 0.05	11.69 ± 1.71	2.52 ± 0.05	19.56 ± 2.31
**7**	1.64 ± 0.16	18.64 ± 1.22	6.85 ± 0.31	29.73 ± 2.78
β-Lap	4.50 ± 0.32	5.85 ± 0.62	4.89 ± 0.36	8.12 ± 0.51
Cisplatin	5.96 ± 0.60	20.91 ± 1.78	2.92 ± 0.01	20.43 ± 1.12

**Table 7:** The scoring values (ΔG) obtained for compounds **1** and **5**–**7**.

Ligand	ΔG [kcal/mol]
**1**	−8.1
**5**	−9.5
**6**	−10.0
**7**	−9.2

## Data Availability

The data presented in this study are available from the authors.

## Supplementary Materials

The following supporting information can be downloaded at: https://www.mdpi.com/article/10.3390/ijms26115331/s1.
