# Supplementary material for: The 8-Hydroxyquinoline Derivatives of 1,4-Naphthoquinone: Synthesis, Computational Analysis, and Anticancer Activity
Source: Int J Mol Sci. 2025 Jun 1;26(11):5331. doi: 10.3390/ijms26115331 (PMC12154095; doi:10.3390/ijms26115331)
Supplement: Supplementary file 1 [file ijms-26-05331-s001.zip › ijms-3618360-supplementary.pdf]

Supplementary materials

The aryl derivatives of 1,4-naphthoquinone: synthesis, computational analysis and anticancer activity

Arkadiusz Sokal, Roman Wrzalik, Małgorzata Latocha, Monika Kadela-Tomanek

Table of contents

Figure S1. The HR-MS spectrum of 2-(8-hydroxyquinolin-5-yl)-1,4-naphthalenedione **5** 2
Figure S2. The HR-MS spectrum of 2-(8-hydroxy-2-methylquinolin-5-yl)-1,4-naphthalenedione **6** 2
Figure S3. The HR-MS spectrum of 2-(8-hydroxy-2-morpholinoquinolin-5-yl)-1,4-naphthalenedione **7** 3
Figure S4. The ^{1}H NMR spectrum of 2-(8-hydroxyquinolin-5-yl)-1,4-naphthalenedione **5** 3
Figure S5. The ^{13}C NMR spectrum of 2-(8-hydroxyquinolin-5-yl)-1,4-naphthalenedione **5** 4
Figure S6. The ^{1}H-^{1}H ROESY spectrum of 2-(8-hydroxyquinolin-5-yl)-1,4-naphthalenedione **5** 4
Figure S7. The ^{1}H-^{13}C HSQC spectrum of 2-(8-hydroxyquinolin-5-yl)-1,4-naphthalenedione **5** 5
Figure S8. The ^{1}H-^{13}C HMBC spectrum of 2-(8-hydroxyquinolin-5-yl)-1,4-naphthalenedione **5** 5
Figure S9. The ^{1}H NMR spectrum of 2-(8-hydroxy-2-methylquinolin-5-yl)-1,4-naphthalenedione **6** 6
Figure S10. The ^{13}C NMR spectrum of 2-(8-hydroxy-2-methylquinolin-5-yl)-1,4-naphthalenedione **6** ... 6
Figure S11. The ^{1}H-^{1}H ROESY spectrum of 2-(8-hydroxy-2-methylquinolin-5-yl)-1,4-naphthalenedione **6** 7
Figure S12. The ^{1}H-^{13}C HSQC spectrum of 2-(8-hydroxy-2-methylquinolin-5-yl)-1,4-naphthalenedione **6** 7
Figure S13. The ^{1}H-^{13}C HMBC spectrum of 2-(8-hydroxy-2-methylquinolin-5-yl)-1,4-naphthalenedione **6** 8
Figure S14. The ^{1}H NMR spectrum of 2-(8-hydroxy-2-morpholinoquinolin-5-yl)-1,4-naphthalenedione **7** 8
Figure S15. The ^{13}C NMR spectrum of 2-(8-hydroxy-2-morpholinoquinolin-5-yl)-1,4-naphthalenedione **7** 9
Figure S16. The ^{1}H-^{1}H ROESY spectrum of 2-(8-hydroxy-2-morpholinoquinolin-5-yl)-1,4-naphthalenedione **7** 9
Figure S17. The ^{1}H-^{13}C HSQC spectrum of 2-(8-hydroxy-2-morpholinoquinolin-5-yl)-1,4-naphthalenedione **7** 10
Figure S18. The ^{1}H-^{13}C HMBC spectrum of 2-(8-hydroxy-2-morpholinoquinolin-5-yl)-1,4-naphthalenedione **7** 10
Table S1. The chemical shift of compound **6** 11
Table S2. The chemical shift of compound **7** 12
Table S3. Interaction of ligand **5-7** with active site of NQO1 protein. 13

Figure S1. The HR-MS spectrum of 2-(8-hydroxyquinolin-5-yl)-1,4-naphthalenedione **5**

Mass Spectrum List Report

Analysis Info

Analysis Name	D:\Data\MKT209.d	Acquisition Date	11/15/2024 12:22:10 PM
Method	low_mass_positive.m	Operator	KM
Sample Name	Tune_neg_high_post	Instrument	impact II 1825265.10082
Comment			

Acquisition Parameter

Source Type	ESI	Ion Polarity	Positive	Set Nebulizer	0.3 Bar
Focus	Active	Set Capillary	4000 V	Set Dry Heater	200 °C
Scan Begin	100 m/z	Set End Plate Offset	-500 V	Set Dry Gas	3.0 l/min
Scan End	1000 m/z	Set Charging Voltage	2000 V	Set Divert Valve	Source
		Set Corona	0 nA	Set APCI Heater	0 °C

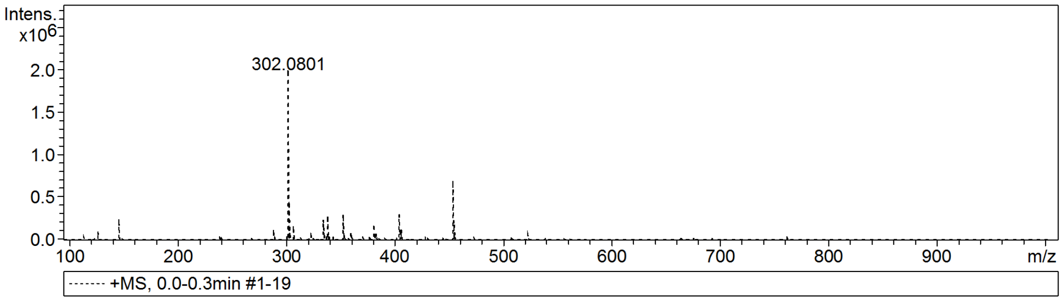

#	m/z	Res.	S/N	I	I %	FWHM
1	302.0801	31189	12720.8	1941351	100.0	0.0097
2	303.0834	26031	2644.3	405939	20.9	0.0116

Figure S2. The HR-MS spectrum of 2-(8-hydroxy-2-methylquinolin-5-yl)-1,4-naphthalenedione **6**

Mass Spectrum List Report

Analysis Info

Analysis Name	D:\Data\test_lc_ms\MKT 208c.d	Acquisition Date	6/18/2024 9:38:17 AM
Method	low_mass_positive.m	Operator	KM
Sample Name	cf3	Instrument	impact II 1825265.10082
Comment			

Acquisition Parameter

Source Type	ESI	Ion Polarity	Positive	Set Nebulizer	0.3 Bar
Focus	Active	Set Capillary	4000 V	Set Dry Heater	200 °C
Scan Begin	100 m/z	Set End Plate Offset	-500 V	Set Dry Gas	3.0 l/min
Scan End	1000 m/z	Set Charging Voltage	2000 V	Set Divert Valve	Source
		Set Corona	0 nA	Set APCI Heater	0 °C

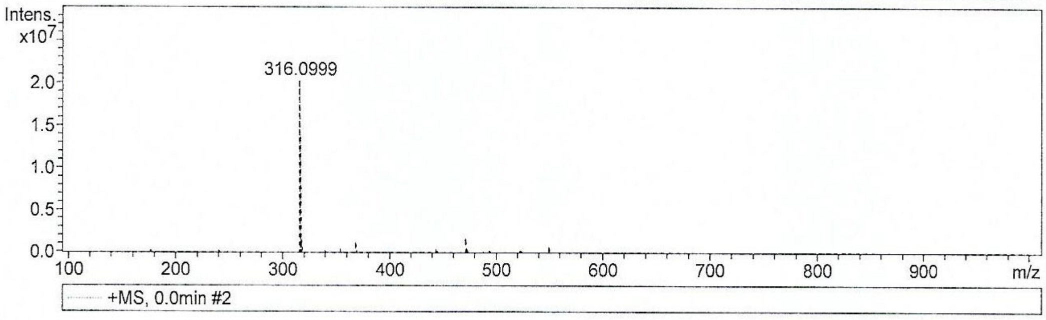

#	m/z	Res.	S/N	I	I %	FWHM
1	316.0999	11103	79526.4	20377596	100.0	0.0285
2	317.1003	39324	54228.4	13951822	68.5	0.0081

Figure S3. The HR-MS spectrum of 2-(8-hydroxy-2-morpholinoquinolin-5-yl)-1,4-naphthalenedione **7**

Mass Spectrum List Report

Analysis Info

Analysis Name	D:\Data\test_lc_ms\MKT 237.d	Acquisition Date	6/18/2024 9:44:58 AM
Method	low_mass_positive.m	Operator	KM
Sample Name	cf3	Instrument	impact II 1825265.10082
Comment			

Acquisition Parameter

Source Type	ESI	Ion Polarity	Positive	Set Nebulizer	0.3 Bar
Focus	Active	Set Capillary	4000 V	Set Dry Heater	200 °C
Scan Begin	100 m/z	Set End Plate Offset	-500 V	Set Dry Gas	3.0 l/min
Scan End	1000 m/z	Set Charging Voltage	2000 V	Set Divert Valve	Source
		Set Corona	0 nA	Set APCI Heater	0 °C

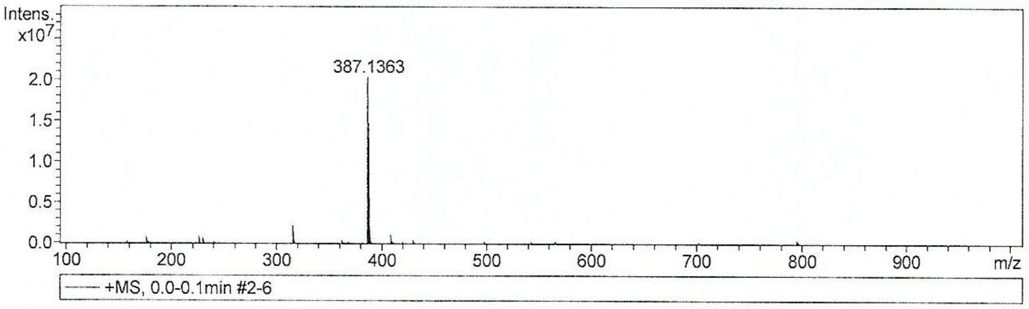

#	m/z	Res.	S/N	I	I %	FWHM
1	387.1363	12562	73499.4	20377596	100.0	0.0308
2	388.1365	42915	52715.8	14649265	71.9	0.0090

Figure S4. The ^{1}H NMR spectrum of 2-(8-hydroxyquinolin-5-yl)-1,4-naphthalenedione **5**

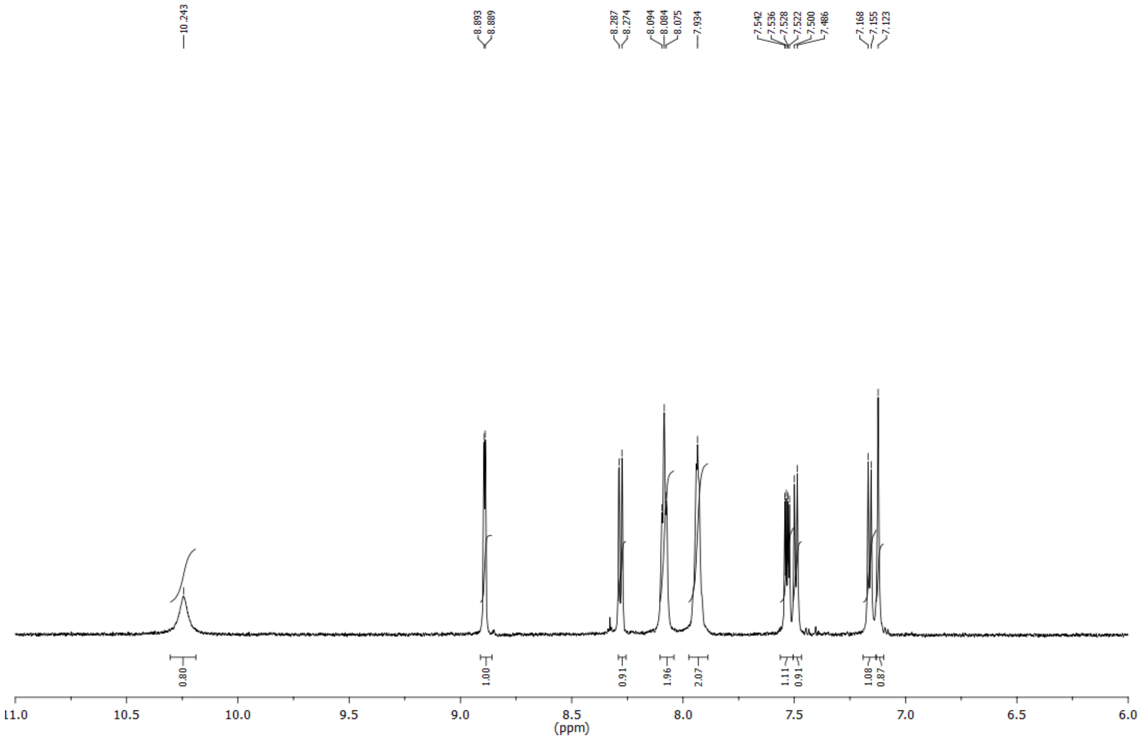

Figure S5. The ^{13}C NMR spectrum of 2-(8-hydroxyquinolin-5-yl)-1,4-naphthalenedione **5**

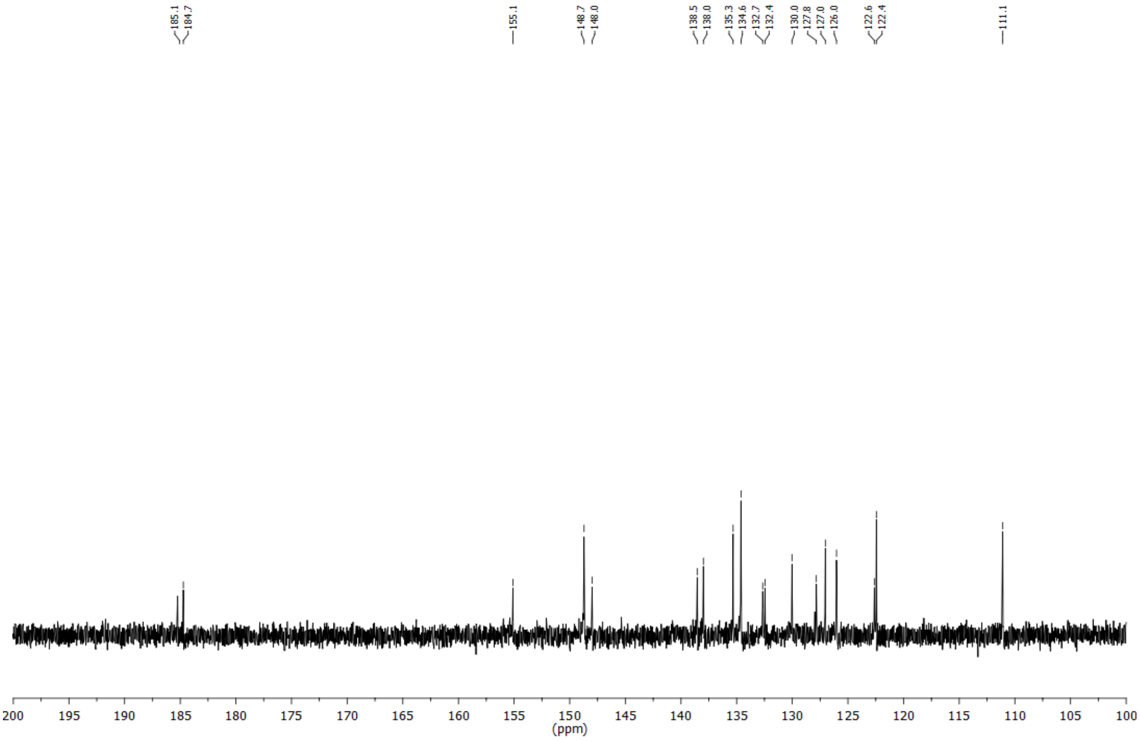

Figure S6. The ^{1}H-^{1}H ROESY spectrum of 2-(8-hydroxyquinolin-5-yl)-1,4-naphthalenedione **5**

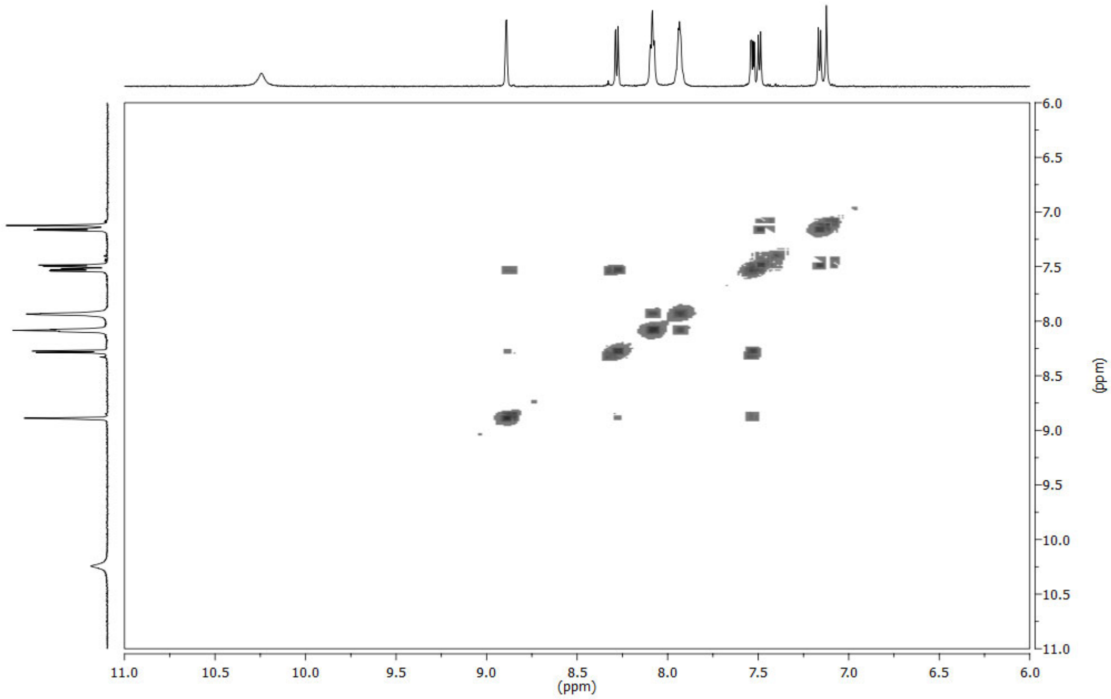

Figure S7. The ^{1}H-^{13}C HSQC spectrum of 2-(8-hydroxyquinolin-5-yl)-1,4-naphthalenedione **5**

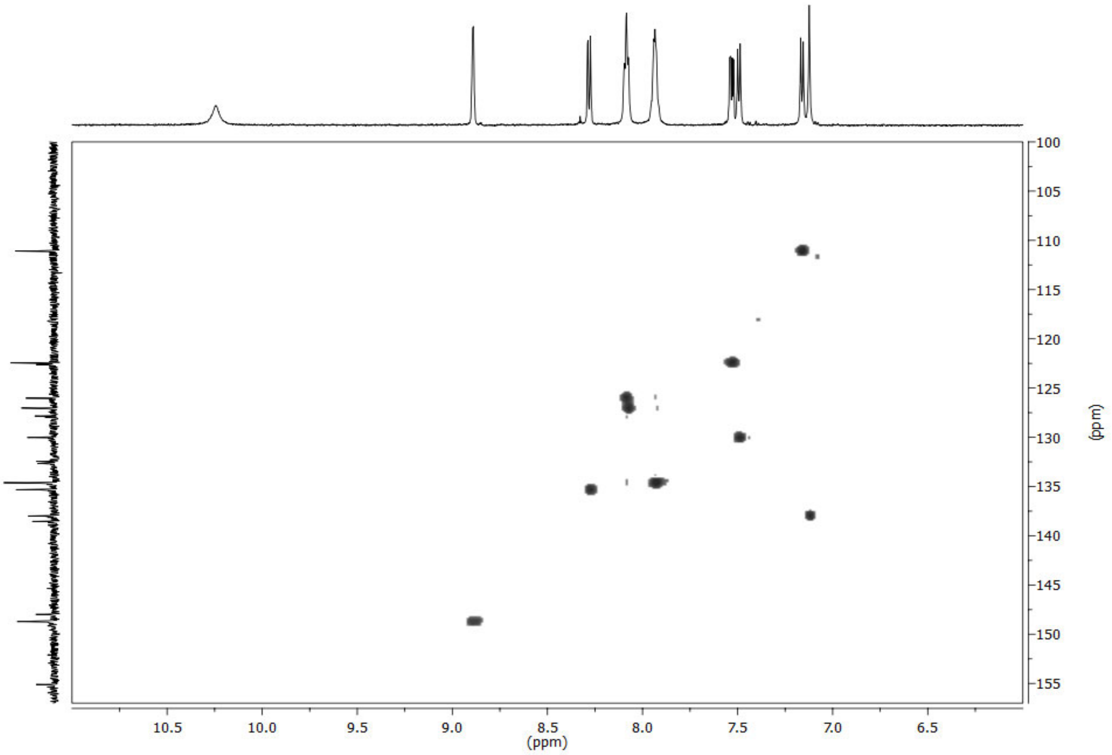

Figure S8. The ^{1}H-^{13}C HMBC spectrum of 2-(8-hydroxyquinolin-5-yl)-1,4-naphthalenedione **5**

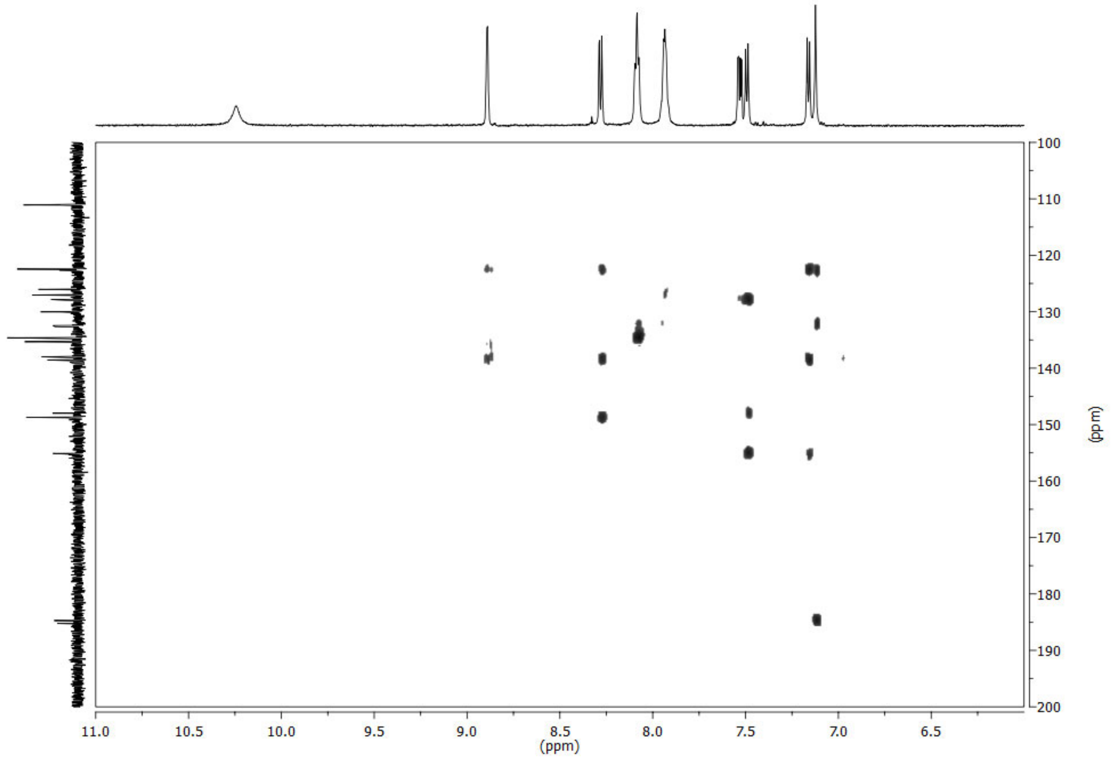

Figure S9. The ¹H NMR spectrum of 2-(8-hydroxy-2-methylquinolin-5-yl)-1,4-naphthalenedione **6**

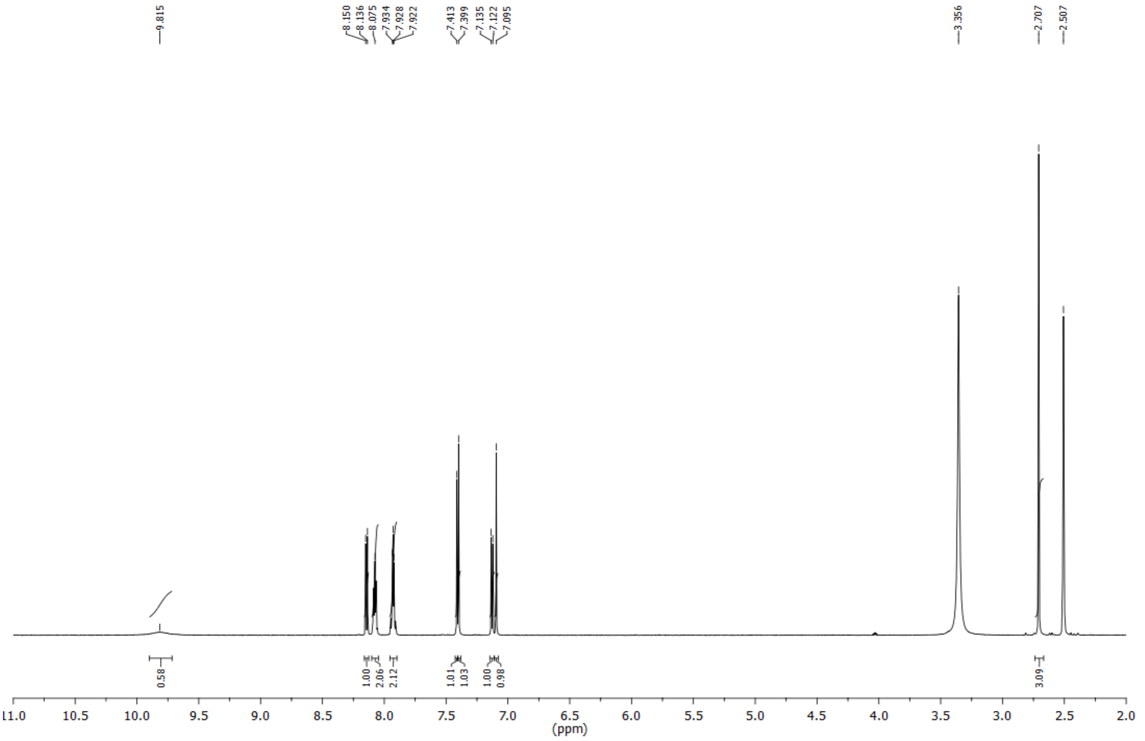

Figure S10. The ¹³C NMR spectrum of 2-(8-hydroxy-2-methylquinolin-5-yl)-1,4-naphthalenedione **6**

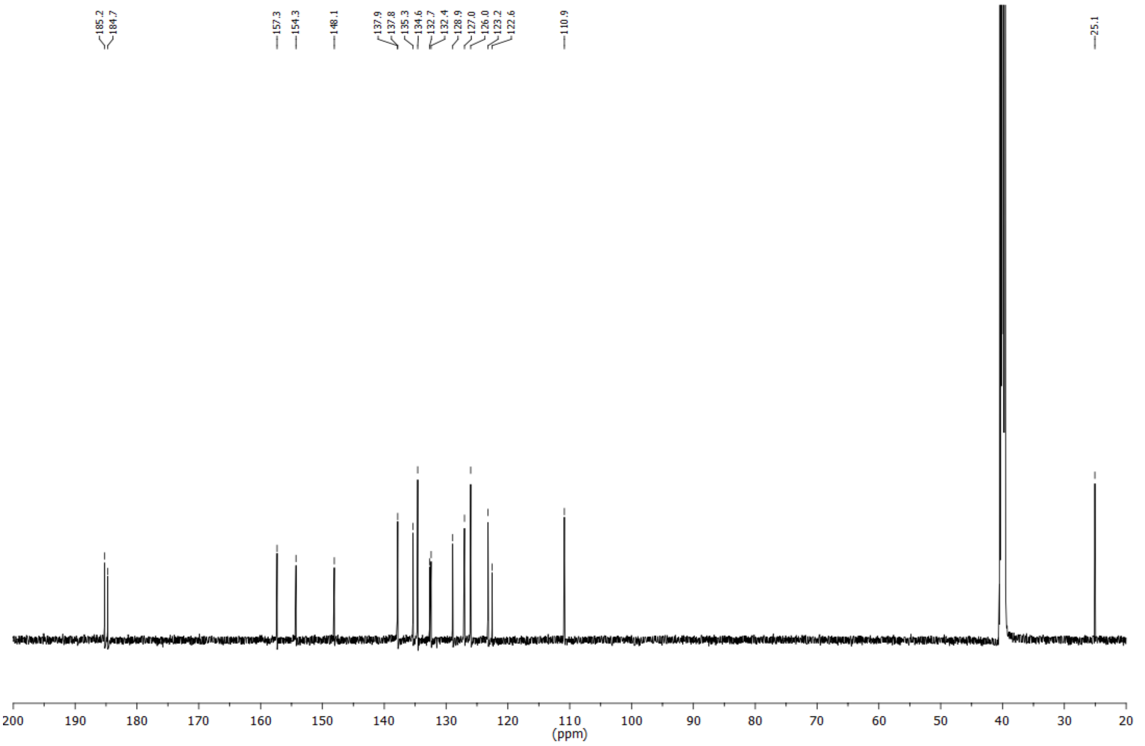

Figure S11. The ^{1}H-^{1}H ROESY spectrum of 2-(8-hydroxy-2-methylquinolin-5-yl)-1,4-naphthalenedione **6**

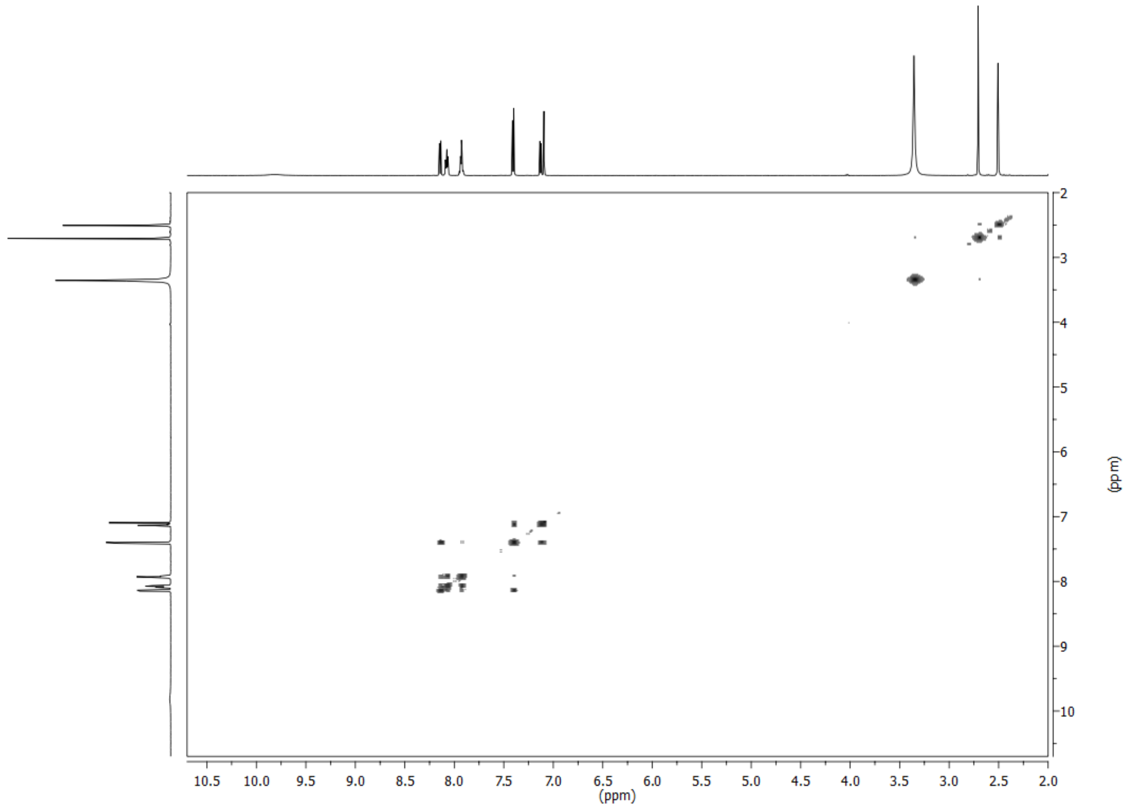

Figure S12. The ^{1}H-^{13}C HSQC spectrum of 2-(8-hydroxy-2-methylquinolin-5-yl)-1,4-naphthalenedione **6**

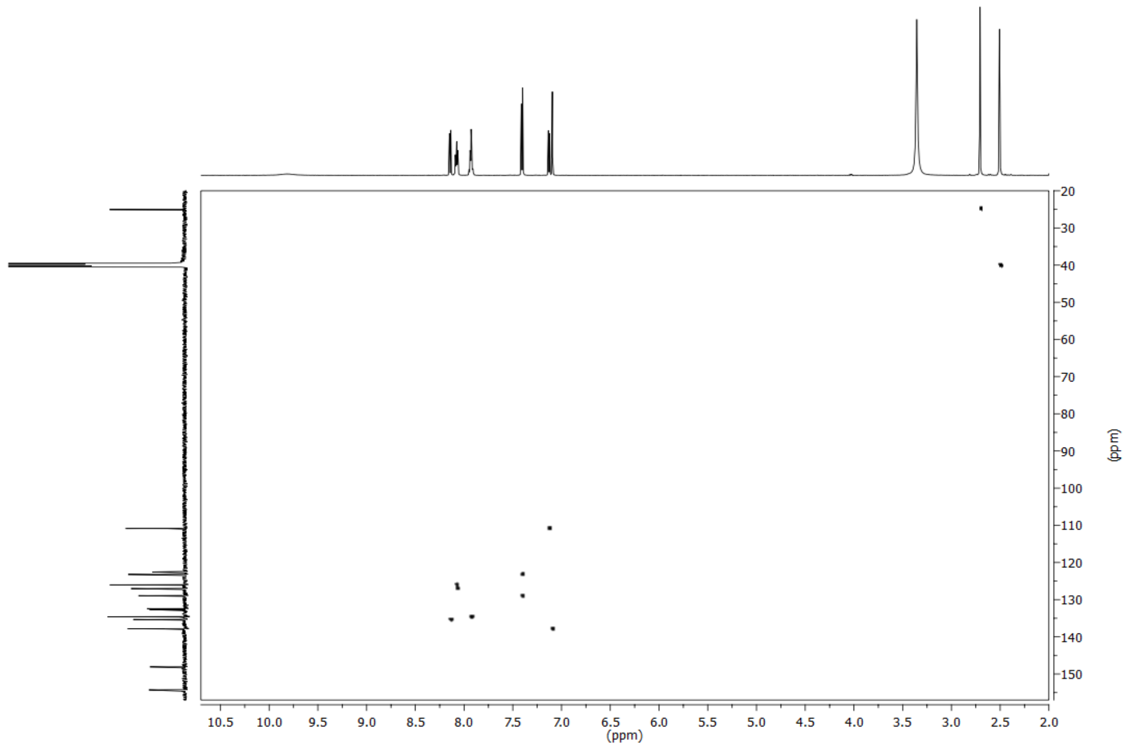

Figure S13. The ^{1}H-^{13}C HMBC spectrum of 2-(8-hydroxy-2-methylquinolin-5-yl)-1,4-naphthalenedione **6**

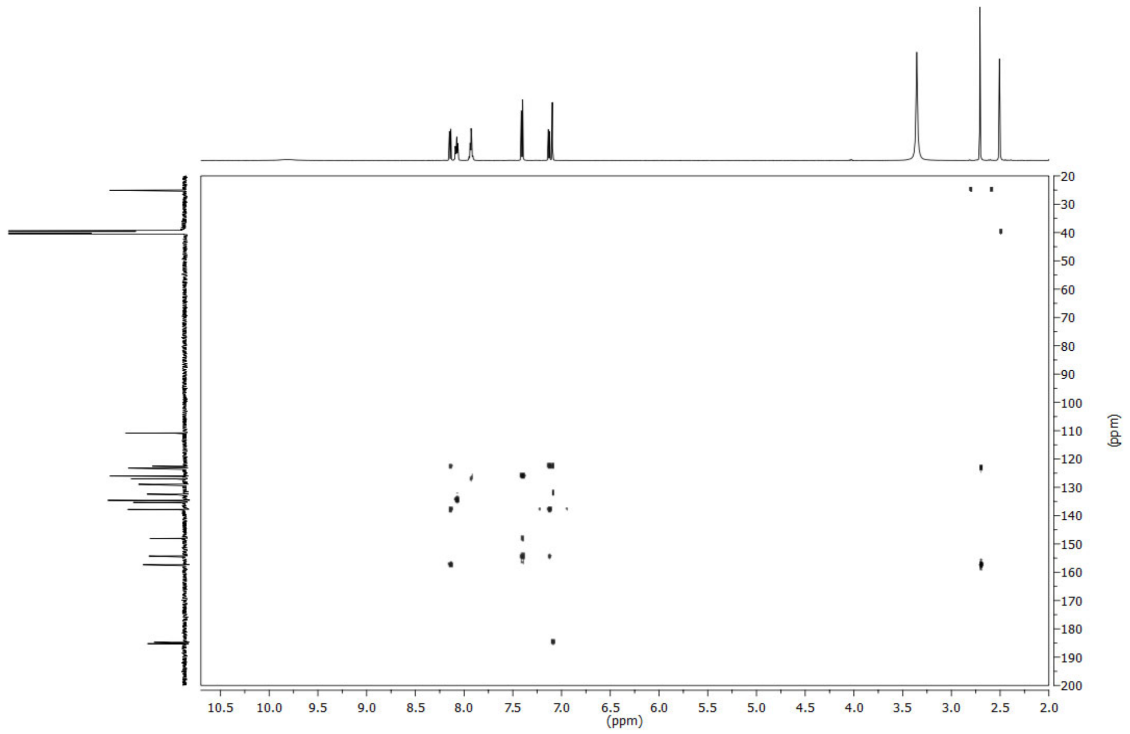

Figure S14. The ^{1}H NMR spectrum of 2-(8-hydroxy-2-morpholinoquinolin-5-yl)-1,4-naphthalenedione **7**

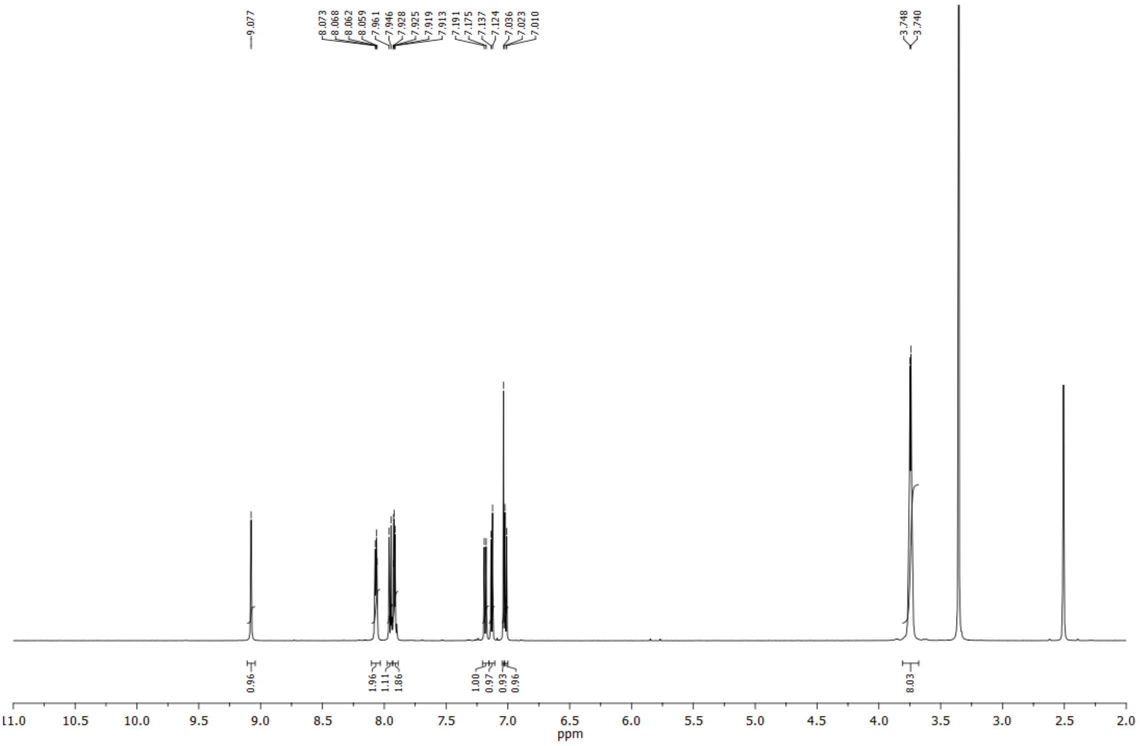

Figure S15. The ^{13}C NMR spectrum of 2-(8-hydroxy-2-morpholinoquinolin-5-yl)-1,4-naphthalenedione **7**

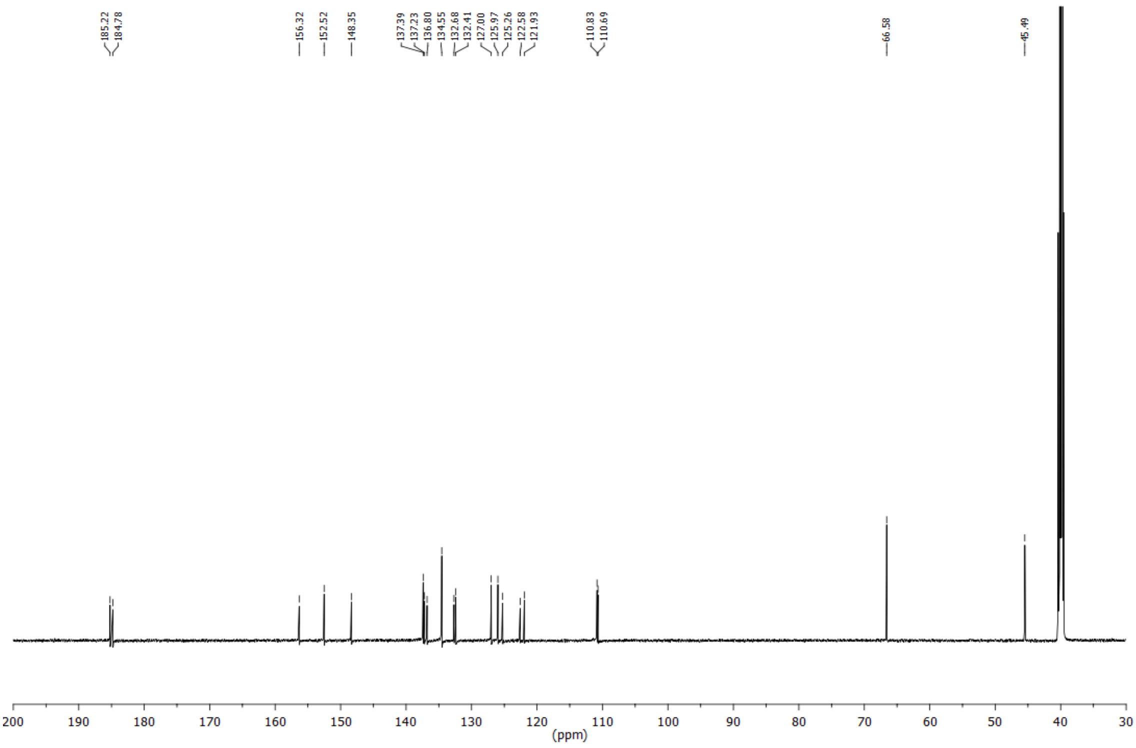

Figure S16. The ^{1}H-^{1}H ROESY spectrum of 2-(8-hydroxy-2-morpholinoquinolin-5-yl)-1,4-naphthalenedione **7**

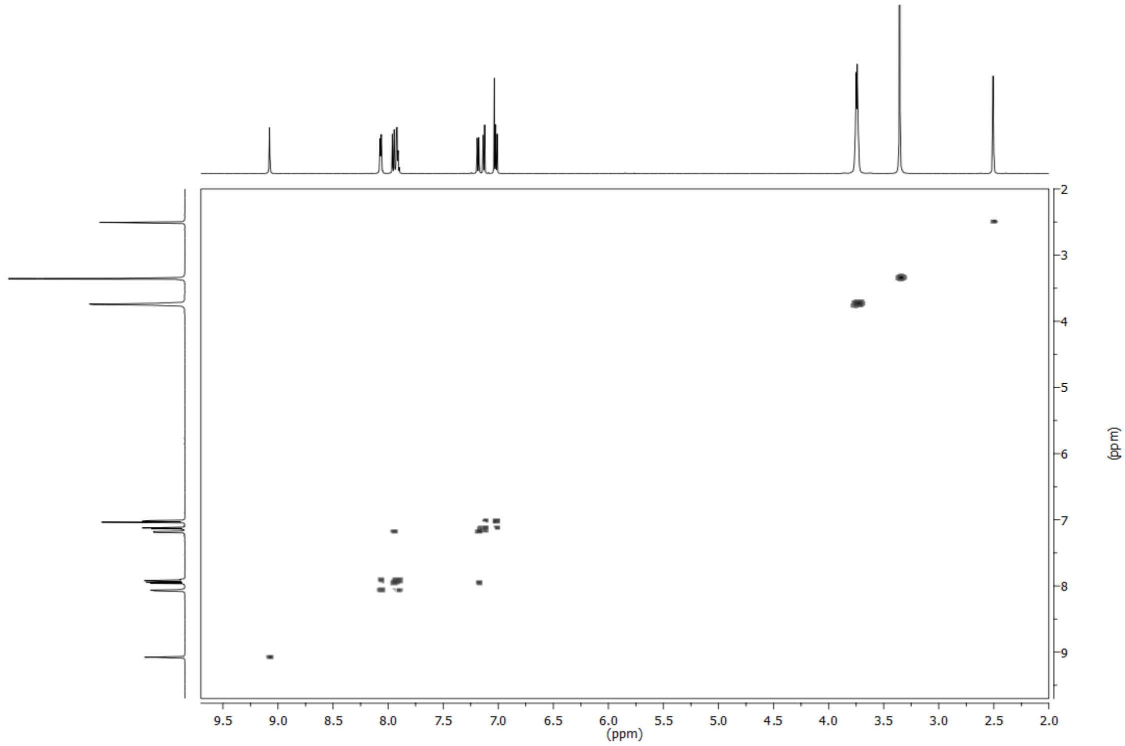

Figure S17. The ^{1}H-^{13}C HSQC spectrum of 2-(8-hydroxy-2-morpholinoquinolin-5-yl)-1,4-naphthalenedione **7**

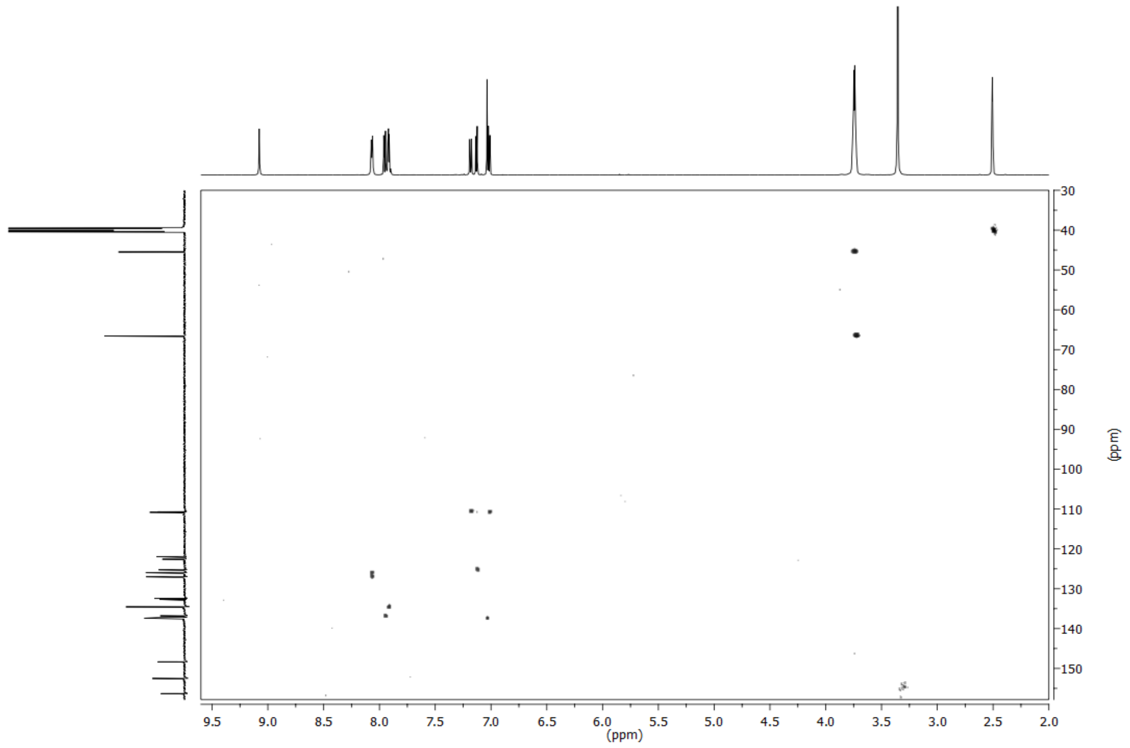

Figure S18. The ^{1}H-^{13}C HMBC spectrum of 2-(8-hydroxy-2-morpholinoquinolin-5-yl)-1,4-naphthalenedione **7**

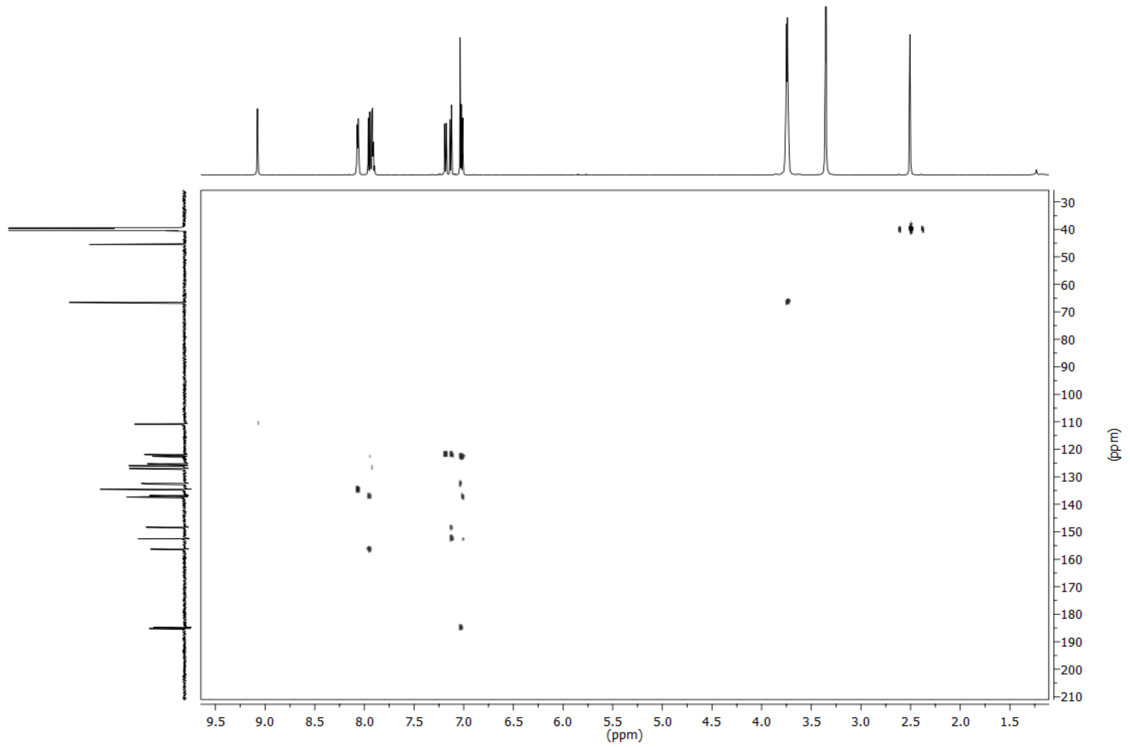

Table S1. The chemical shift of compound **6**.

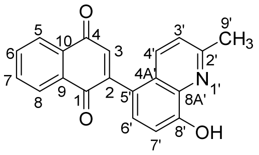

Proton	^{1}H NMR [ppm]	ROESY	HSQC	Carbon	^{13}C NMR [ppm]	HMBC
OH	9.82					
H4′	8.15	H6′(7.40)-H9′(2.71)	H6′(7.40)-C4′(135.3)	C4′	135.3	H6′(7.40)-C3(137.8)
						H6′(7.40)-C2′(157.3)
						H6′(7.40)- C4A′(122.6)
						H6′(7.40)-C8′(154.3)
H5	8.07	H5/H8(8.07)-H6/H7(7.93)	H5/H8(8.07)-C5(126.0)	C5	126.0	H5/H8(8.07)-C6(134.6)
H8			H5/H8(8.07)-C8(127.0)	C8	127.0	H5/H8(8.07)-C1(185.2)
						H5/H8(8.07)-C4(184.7)
						H5/H8(8.07)-C10(132.4)
H6	7.93	-	H6/H7(7.93)-C6/C7(134.6)	C6	134.6	H6/H7(7.93)-C5(126.0)
H7				C7		H6/H7(7.93)-C8(127.0)
						H6/H7(7.93)- C10(132.4)
						H6/H7(7.93)-C9(132.7)
H3′	7.41	H3′(7.41)-H9′(2.71)	H3′(7.41)-C3′(123.2)	C3′	123.2	H3′(7.41)-C9′(25.1)
						H3′(7.41)-C2′(157.3)
H6′	7.40	H6′(7.40)-H7′(7.14)	H6′(7.40)-C6′(128.9)	C6′	128.9	H6′(7.40)-C5′(126.1)
						H6′(7.40)-C2(148.1)
						H6′(7.40)-C8′(154.3)
						H6′(7.40)-C7′(110.9)
H7′	7.14	-	H7′(7.14)-C7′(110.9)	C7′	110.9	H7′(7.14)- C4A′(122.6)
						H7′(7.14)- C8A′(137.8)
						H7′(7.14)-C8′(154.3)
						H7′(7.14)-C2′(157.3)
H3	7.09	-	H3(7.09)-C3(137.8)	C3	137.8	H3(7.09)-C4 (184.7)
						H3(7.09)-C2 (148.1)
						H3(7.09)-C10(132.4)
						H3(7.09)-C4A′(122.6)
H9′	2.71	-	H9′(2.71)-C9′(25.1)	C9′	25.1	H9′(2.71)-C3′(123.2)
						H9′(2.71)-C7′(110.9)
						H9′(2.71)-C2′(157.3)
						H9′(2.71)-C3(137.8)
				C2′	157.3	C2′(157.3)-H4′(8.15)
						C2′(157.3)-H3′(7.41)
						C2′(157.3)-H7′(7.14)
						C2′(157.3)-H9′(2.71)
				C4A′	122.6	C4A′(122.6)-H4′(8.15)
						C4A′(122.6)-H7′(7.14)
						C4A′(122.6)-H3(7.09)
				C5′	126.1	C5′(126.1)-H3(7.09)
						C5′(126.1)-H6′ (7.40)
				C10	132.4	C10(132.4)-H5/H8(8.07)
						C10(132.4)-H6/H7(7.93)
						C10(132.4)-H3(7.09)

				C9	132.7	C9(132.7)-H6/H7(7.93)
				C8A′	137.8	C8A′(137.8)-H7′(7.14)
						C8A′(137.8)-H9′(2.71)
				C2	148.1	C2(148.1)-H6′(7.40)
						C2(148.1)-H3(7.09)
				C8′	154.3	C8′(154.3)-H4′(8.15)
						C8′(154.3)-H6′(7.40)
						C8′(154.3)-H7′(7.14)
				C4	184.7	C4(184.7)-H5/H8(8.07)
						C4(184.7)-H3(7.09)
				C1	185.2	C1(185.2)-H5/H8(8.07)

Table S2. The chemical shift of compound **7**.

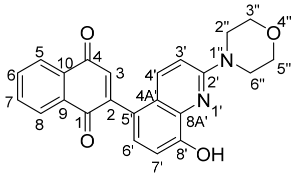

Proton	^{1}H NMR [ppm]	ROESY	HSQC	Carbon	^{13}C NMR [ppm]	HMBC
OH	9.08					
H4′	7.96	H4′(7.96)-H3′(7.19)	H4′(7.96)-C4′(136.8)	C4′	136.8	H4′(7.96)- C4A′(122.6)
						H4′(7.96)- C8A′(137.2)
						H4′(7.96)-C2′(156.3)
H5	8.07	H5/H8(8.07)-	H5/H8(8.07)-C5(126.0)	C5	126.0	H5/H8(8.07)-C6(134.5)
H8		H6/H7(7.92)	H5/H8(8.07)-C8(127.0)	C8	127.0	H5/H8(8.07)-C10(132.4)
H6	7.92	-	H6/H7(7.92)-C6/C7(134.5)	C6	134.5	H6/H7(7.92)-C8(127.0)
H7				C7		
H3′	7.19	H3′(7.19)-H6′(7.14)	H3′(7.19)-C3′(110.7)	C3′	110.7	H3′(7.19)-C5′(121.9)
						H3′(7.19)-C2′(156.3)
H6′	7.14	H6′(7.14)-H7′(7.02)	H6′(7.14)-C6′(125.3)	C6′	125.3	H6′(7.14)-C5′(121.9)
						H6′(7.14)-C2(148.3)
						H6′(7.14)-C8′(152.7)
H3	7.04	-	H3(7.04)-C3(110.8)	C3	110.8	H3(7.04)- C4A′(122.6)
						H3(7.04)-C9(132.7)
						H3(7.04)-C4(184.8)
H7′	7.02	-	H7′(7.02)-C7′(137.4)	C7′	137.4	H7′(7.02)- C4A′(122.6)
						H7′(7.02)- C8A′(137.2)
						H7′(7.02)-C8′(152.7)
H2′′-H6′′	3.75	H2′′(3.75)-H3′(7.19)	H2′′(3.75)-C5′′(66.6)	C3′′/C5′′	45.5	H2′′(3.75)- C6′′(66.6)
			H3′′(3.75)- C6′′(45.5)	C2′′/C6′′	66.6	
				C8′	152.7	C8′(152.7)-H6′(7.14)
				C5′	121.9	C5′(121.9)-H3′(7.19)
						C5′(121.9)-H6′(7.14)
				C4A′	122.6	C4A′(122.6)-H4′(7.96)
						C4A′(122.6)-H7′(7.02)
						C4A′(122.6)-H3(7.04)
				C10	132.4	C10(132.4)-H5/H8(8.07)
				C9	132.7	C9(132.7)-H3(7.04)

C8A′	137.2	C8A′(137.2)-H4′(7.96)
		C8A′(137.2)-H7′(7.02)
C2	148.3	C2(148.3)-H6′(7.14)
C2′	156.3	C2′(156.3)-H4′(7.96)
		C2′(156.3)-H3′(7.19)
C4	184.8	C4(184.8)-H3(7.04)
C1	185.2	-

Table S3. Interaction of ligand **5-7** with active site of NQO1 protein.

Ligand	H-bonding residues and length (Å)	π-interaction residues and length (Å)
**5**	TYR128 (2,18; 2,40)<br>HIS161 (2,76)<br>HIS194 (3,04)	TRP105 (4,53)<br>PHE178 (4,48; 5,24)<br>FAD (4,03; 3,75; 4,13; 4,51; 5,44)
**6**	TYR128 (2,18; 2,40)<br>HIS161 (2,74)<br>HIS194 (3,05)	TRP105 (4,53)<br>GLY149 (3,83)<br>PHE178 (5,23; 4,46)<br>HIS194 (4,26)<br>PHE232 (5,17)<br>FAD (3,75; 4,04; 4,53; 4,14; 5,47)
**7**	TYR126 (2,39)<br>GLY193 (2,26)<br>HIS194 (2,54)	TRP105 (5,01)<br>GLY149 (3,66; 4,30)<br>PHE178 (5,72; 4,02)<br>HIS194 (5,00)<br>FAD (3,58; 4,04; 3,99; 4,59; 5,58)

**14b**	TYR128 (2.061)	TYR128 (4.344; 3.572; 5.104; 4.488)<br>TYR126 (4.675)<br>PHE178 (5.807; 5.126)<br>PHE236 (4.688)<br>TRP105 (5.800; 5.245)<br>MET154 (4.468; 5.251)<br>HIS161 (4.340)<br>FAD (3.780; 4.016; 4.602; 3.818; 4.992)
